# Bisphenazine-Based Porous Aromatic Frameworks for Photocatalytic and Electrocatalytic Oxygen Activation

**DOI:** 10.1021/acsanm.6c02207

**Published:** 2026-07-29

**Authors:** Miguel Sánchez-Fuente, Juan Manuel Hernández-Gómez, Julia Arnanz, Víctor Cepa-López, Sonia Bruña, Emiliano Martínez-Periñán, Alicia Moya, Inés Corral, Cristina Gutiérrez-Sánchez, Rubén Mas-Ballesté

**Affiliations:** † Department of Inorganic Chemistry (Module 7), Facultad de Ciencias, Universidad Autónoma de Madrid, 28049 Madrid, Spain; ‡ Department of Analytical Chemistry (Module 16), Facultad de Ciencias, Universidad Autónoma de Madrid, 28049 Madrid, Spain; § Department of Chemistry (Module 13), Facultad de Ciencias, Universidad Autónoma de Madrid, 28049 Madrid, Spain; ∥ Institute for Advanced Research in Chemical Sciences (IAdChem), Universidad Autónoma de Madrid, 28049 Madrid, Spain

**Keywords:** photocatalysis, organic materials, porous aromatic frameworks, electrocatalysis, oxygen reduction reaction, oxygen activation

## Abstract

Two closely related bisphenazine-based porous aromatic frameworks (PAFs), incorporating either phenyl (PAF-Phen) or benzothiadiazole (PAF-Phen-BT) linkers, were synthesized and investigated as metal-free nanostructured materials for photocatalytic and electrocatalytic oxygen activation. Both frameworks were comprehensively characterized using spectroscopic, structural, and surface techniques, and their optical and electronic properties were evaluated. The photocatalytic performance of the materials was assessed in aerobic oxidation reactions under visible-light irradiation, while their electrocatalytic activity toward the oxygen reduction reaction (ORR) was examined under electrochemical conditions. Density functional theory calculations were performed to characterize the excited-state properties underlying the photocatalytic behavior and to compute the vertical ionization energies, electron attachment energies, and O_2_ adsorption energies related to the electrocatalytic activity. The combined experimental and computational results show that PAF-Phen exhibits superior photocatalytic activity, operating predominantly through energy-transfer-mediated photosensitization of molecular oxygen, whereas PAF-Phen-BT displays enhanced electrocatalytic performance toward oxygen reduction. These contrasting behaviors are associated with framework-dependent electronic structure, excited-state dynamics, and oxygen–material interactions, which selectively bias the materials toward energy-transfer or electron-transfer-dominated pathways.

## Graphical abstract

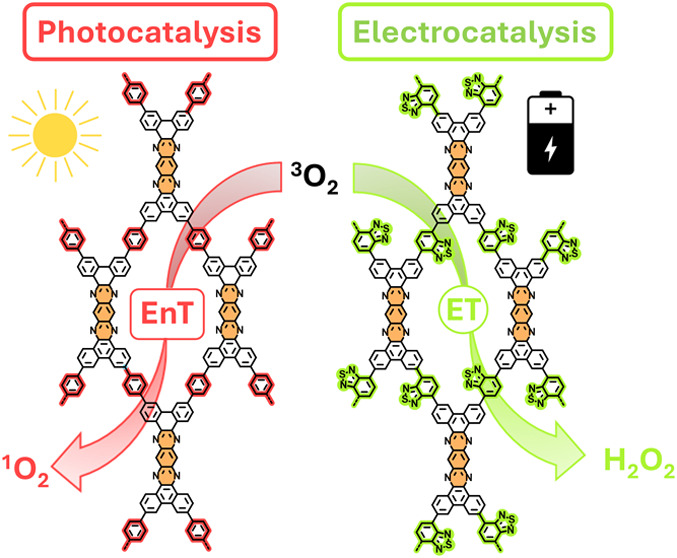

## Introduction

The development of metal-free catalytic materials capable of promoting photocatalytic and/or electrocatalytic transformations has attracted increasing attention in recent years, driven by sustainability concerns and the need for earth-abundant alternatives to noble metal systems.^1,2^ In this context, organic semiconductors and in particular, porous aromatic frameworks (PAFs) have emerged as promising platforms for both photocatalytic and electrocatalytic applications^3,4^ due to their structural tunability, chemical stability, and modular electronic properties. Their extended π-conjugated networks enable visible-light absorption, while the incorporation of heteroatoms and donor–acceptor units allows fine control over band gaps, charge transport, and interfacial interactions.^5,6^ As a result, these nanostructured materials have been explored in applications ranging from photocatalytic to electrocatalytic processes such as pollutant degradation, aerobic oxidation, or oxygen reduction reaction (ORR) among others.^6–10^

A key challenge in the rational design of organic catalytic materials lies in understanding and controlling the mechanistic pathways that govern their activity under different operating conditions. Photocatalytic processes in organic semiconductor materials can proceed via energy-transfer (EnT) and/or electron-transfer (ET) pathways, which often operate simultaneously.^8,11^ In contrast, electrocatalytic reactions are inherently governed by electron-transfer processes but follow distinct kinetic and thermodynamic principles, as they are controlled by the applied potential, interfacial charge transport, and adsorbate–surface interactions.^12^ Despite these fundamental differences, photo- and electrocatalytic activities are frequently rationalized using similar descriptors, including band gaps, frontier orbital energies, valence and conduction band positions, heteroatom content, charge distribution, and charge carrier mobility.^12–14^ Although these parameters are useful for describing the general electronic structure of materials, they do not univocally discriminate between excited-state energy-transfer processes and interfacial electron-transfer reactions. Consequently, structure–activity relationships governing photocatalysis and electrocatalysis are often implicitly assumed to be transferable, even though the underlying mechanisms may substantially differ. Experimental systems that enable the selective identification and decoupling of EnT-dominated photocatalysis from ET-driven electrocatalysis are therefore highly valuable for elucidating the factors that direct photo- and electrocatalytic activity in porous organic materials.

A widely used approach to endow porous organic materials with photo- and/or electrocatalytic functionality involves incorporating extended aromatic motifs bearing heteroatoms into the organic framework. Nitrogen-rich polycyclic aromatic motifs, such as phenazine and bisphenazine units, have attracted sustained interest in photo- and electrocatalytic materials due to their reversible redox activity, extended conjugation, and ability to access long-lived excited states.^15,16^ Phenazines have been extensively studied as redox mediators and electron shuttles in both biological and synthetic systems. They typically undergo highly reversible two-electron/two-proton (2e^–^/2H^+^) redox processes in aqueous media, and their functionalized derivatives, such as those substituted at the 2,3 positions, exhibit remarkable chemical stability and resistance to undesired side reactions.^17^ In addition, phenazines are well-known chromophores capable of photosensitizing molecular oxygen through triplet-state energy transfer.^18^ More recently, phenazine-based motifs have been incorporated into extended organic frameworks to enhance charge transport, oxygen activation, and light-harvesting properties.^15,19^ Despite this progress, most previous studies have addressed photocatalysis or electrocatalysis independently, often assuming that similar structural features govern both the activities. Direct experimental evidence demonstrating how closely related phenazine-based frameworks can selectively favor EnT-driven photocatalysis or ET-mediated electrocatalysis within the same structural platform remains unexplored. Consequently, a detailed understanding of the structural features that control photo- and/or electrocatalytic activity in phenazine-based organic materials would provide a powerful tool for the rational design of new catalytic systems with predetermined functions.

In this work, we address this gap by synthesizing and investigating two closely related bisphenazine-based porous aromatic frameworks, incorporating either phenyl (PAF-Phen) or benzothiadiazole (PAF-Phen-BT) linkers. By combining spectroscopic characterization, photocatalytic and electrocatalytic studies, and density functional theory calculations, we demonstrate that subtle changes in the framework composition are sufficient to invert the dominant oxygen activation pathway. Specifically, one material operates as an efficient photosensitizer for singlet oxygen generation via energy transfer, whereas the other preferentially promotes electron-transfer-mediated oxygen reduction under electrochemical conditions. These results provide mechanistic insight into the distinct principles governing photo- and electrocatalytic activity in organic porous materials and establish bisphenazine-based frameworks as a model platform to disentangle EnT and ET processes through rational structural design.

## Experimental Section

### Synthesis of Bisphenazine Building Units (1)

Into a 250 mL Schlenk flask, 3,6-dibromophenanthrene-9,10-dione (356 mg, 0.97 mmol), benzene-1,2,4,5-tetraamine tetrahydrochloride (138 mg, 0.4856 mmol), and potassium carbonate (335 mg, 2.43 mmol) were added. The flask was sealed with a septum and 3 vacuum–Ar cycles were applied. After that, 40 mL of a previously Ar-purged THF:MeOH (1:1) mixture was added through the septum. Then, the reaction mixture was heated to 75 °C for 12 h under stirring. When the reaction was complete, an intense red-solid precipitate was observed in the crude, which was isolated by centrifugation. The solid was washed with distilled water, MeOH, THF, MeCN, and Et_2_O sequentially, and dried overnight at 70 °C to afford the bisphenazine building unit **1** as a red solid (309 mg, 80% yield).

Due to the high insolubility of building block **Phen-Br**_**4**_ (Scheme S1), its characterization was carried out by FTIR, MALDI-TOF mass spectrometry, and solid-state CP/MAS ^13^C NMR.

#### MALDI-TOF (Negative Mode, DCTB Matrix)

797.8 [M^–^] (theoretical value: 798.13).

#### FTIR (KBr)

1590, 1542, 1499, 1487, 1393, 1343, 1287, 1137, 1094, 1029, 969, 876, 864, 822, 551 cm^–1^.

### Synthesis of PAF-Phen and PAF-Phen-BT

Into a 250 mL Schlenk flask, Phen-Br_4_ **1** (150 mg, 0.17 mmol), Pd­(PPh_3_)_4_ (28.3 mg, 15 mol %), and potassium carbonate (97 mg, 0.70 mmol) were added. For PAF-Phen, the solids were mixed with 1,4-phenylenediboronic acid (58 mg, 0.35 mmol) and for PAF-Phen-BT, with 4,7-bis­(4,4,5,5-tetramethyl-1,3,2-dioxaborolan-2-yl)-2,1,3-benzothiadiazole (146 mg, 0.35 mmol). After that, 3 vacuum–Ar cycles were applied to the flask containing the solids. The flask was sealed with a septum, and 25.2 mL of previously Ar-purged DMF:water (88:12) mixture was added through the septum. Then, the reaction was heated to 140 °C under stirring for 72 h. Finally, the solid was isolated by centrifugation and washed with distilled water, MeOH, DCM, MeCN, and acetone. After drying overnight at 70 °C, PAF-Phen and PAF-Phen-BT were obtained as dark-red solids (91% yield for PAF-Phen and 75% yield for PAF-Phen-BT).

### Photocatalytic Degradation of Para Red

In 12 mL vials, 2 mg of PAF material was dispersed into 10 mL of a 10 ppm para red (PR) solution in a mixture of CH_3_CN/H_2_O (4:1) using an ultrasonication bath for 2 min. The reactions were sealed through a PTFE-rubber septum, and a needle was used to purge with oxygen gas for 2 min. Before the photodegradation tests, adsorption experiments were performed, stirring in dark conditions for 35 min. For the photodegradation experiments, the vials were stirred under the irradiation of blue light LEDs with λ = 465 nm and 18 W/m^2^ for 3 h. Then, the material was collected by filtration, and the supernatants were directly analyzed by UV–vis spectroscopy owing to the intense absorption bands of PR at 483 nm. Negligible degradation was observed when the dye solutions were irradiated in the absence of the photocatalysts. In the mechanistic assays, for the photodegradation experiments under an argon atmosphere, the same general procedure was followed, except that after the reactions were sealed through a PTFE-rubber septum, 3 vacuum freeze–pump–thaw cycles were performed. In the quencher scavenger assays for PR, 5 equiv (0.017 mmol) of the corresponding quencher (1,4-Diazabicyclo [2.2.2]­octane, DABCOor benzoquinone, dissolved in MeOH) with respect to the dye was added.

### Photocatalytic Furfuryl Alcohol (FA) Oxidation Experiments with PAF-Phen and PAF-Phen-BT

In a 12 mL glass vial, PAF-Phen or PAF-Phen-BT (1 mg) was added followed by 3 mL of a 10 mM furfuryl alcohol solution in MeCN. The vial was sealed, purged with O_2_, and sonicated to homogeneously disperse the photocatalyst. Then, the reaction was irradiated (with blue LEDs, λ = 465 nm and 18 W/m^2^) for 1 h under stirring. After this, the crude mixture was filtered and directly analyzed by ^1^H NMR (CDCl_3_) to provide a conversion value by peak integration.

For the singlet oxygen quenching experiments with DABCO, 10.1 mg of DABCO (0.09 mmol, 3 equiv) was added to the reaction mixture and the general procedure described above was followed. For the leaching tests, after 30 min of irradiation, the crude was filtered to remove the PAF-Phen material and mixed up with fresh 10 mM FA solution. Then, the catalyst-free mixture was further irradiated for 1 h. The leaching was evaluated through comparison between the FA/5H5F (5H5F = 5-hydroxy-2­(5H)-furanone) signals ratio of the mixture after the first and second irradiation, obtained by ^1^H NMR integration of spectra. For the recyclability experiments, a slightly higher catalytic loading (3 mg) was employed to compensate for catalyst loss after each cycle. Thus, the CTF-Quin material was isolated by centrifugation, washed with MeCN, and dried at 70 °C.

### Electrocatalytic Studies

Electrochemical experiments were performed using Vionic potentiostat/galvanostat Intello software from Metrohm-Autolab. Glassy carbon electrodes (GCs) were supplied by Pine Instruments. Experiments were run in a three-electrode glass cell using a BAS Ag/AgCl reference electrode (210 mV vs NHE) and a platinum wire counter electrode.

Electrochemical impedance spectroscopy (EIS) measurements were recorded with a sinusoidal potential modulation of ± 5 mV in amplitude superimposed onto the formal potential of the redox probe. EIS experiments were carried out in 0.1 M KCl containing 1.0 × 10^–2^ M ferrocyanide/ferricyanide system (1:1).

### Chemicals

Potassium hydroxide (analytical Carbo Turba), Nafion (Sigma-Aldrich), KCl, K_3_[Fe­(CN)_6_], K_4_[Fe­(CN)_6_], and ethanol (Merck) were prepared by using deionized water obtained from a Millipore Milli-Q purification system.

### Instrumentation and Methods

Electrochemical experiments were performed using a Vionic potentiostat/galvanostat Nova 2.1 software from Metrohm-Autolab. Cyclic voltammetry (CV) was performed at different scan rates: 5, 25, 50, 100, and 150 mV/s. Hydrodynamic linear sweep voltammetry (HLSV) was conducted at 10 mV/s. Both electrochemical measurements were carried out at 0.1 M KOH, pH 13. Electrochemical impedance spectroscopy (EIS) measurements were recorded with a sinusoidal potential modulation of ± 5 mV in amplitude superimposed onto the formal potential of the redox probe. EIS experiments were carried out in 0.1 M phosphate buffer, pH 7.0 in the presence of 0.1 M KCl containing 1.0 × 10^–2^ M ferrocyanide/ferricyanide system (1:1). EIS experiments were performed at the oxygen reduction reaction (ORR) potential in 0.1 M KOH oxygen-saturated conditions.

Electrochemical measurements with no agitation were carried out in a glass and Teflon homemade cell. As working electrodes, glassy carbon electrodes 3 mm of diameter from CH Instruments were used. A homemade saturated calomel electrode (SCE) was used as a reference electrode. A graphite bar electrode was employed as a counter electrode. Hydrodynamic measurements were made using a glassy carbon disk/platinum ring rotating ring disk electrode (RRDE) from PINE and the same counter and reference electrode as in static measurements were employed. Measures were made in a conventional electrochemical cell adapted to RRDE and the speed rotator rate was modulated using MSR Rotator from PINE Instruments. Electrode modification: Glassy carbon electrodes were modified with 10 μL of a suspension made of 20% ethanol, 0.02% Nafion, and 1 mg of each material suspended in Milli-Q water.

### Computational Details

We performed calculations to elucidate experimental observations. The systems were described by using molecular models consisting of PAF-Phen or PAF-Phen-BT tetramers, as shown in Figure 8. All calculations were carried out using the Orca 6.1.0 software package^20^ within the framework of density functional theory and its time-dependent version (TD-DFT). The CAM-B3LYP functional was employed in combination with the def2-SVP basis set^21^ and the corresponding Coulomb-fitted auxiliary basis set,^22^ including D3BJ dispersion corrections.^23,24^ To avoid triplet instabilities, the Tamm–Dancoff approximation was applied throughout.^25^

## Results and Discussion

### Synthesis and Characterization of Materials

The bisphenazine-based building block was synthesized through condensation of a dibromodiketone-phenanthrene with phenyl-tetramine in a 2:1 stoichiometry ratio in a THF:methanol mixture (see experimental part, Scheme S1). Building block **1**, also denoted as Phen-Br_4_, was afforded as a precipitate that was separated by filtration. This building unit presented a low solubility due to the great planarity conferred by the aromatic conjugated rings. For this reason, this building block was characterized by elemental analysis, FTIR, solid-state CP/MAS ^13^C NMR, and MALDI-TOF. The results of this characterization confirmed the successful formation of the bisphenazine cores. FTIR provided useful information about the building unit. Clear signals attributed to the bisphenazine core could be observed. Notably, the C=O (1673 cm^–1^) stretching band from the phenanthrene reactant disappears after the condensation, while the C=N stretching at 1590 cm^–1^ in the phenazine building block appears (see Figure S1). Furthermore, no signals attributed to the tetramine precursor were observed in the building block IR spectrum, demonstrating the efficacy of the condensation (Figure S1). Other significant signals attributed to the phenazine core were observed at 1543, 1343, and 1028 cm^–1^. To further confirm its chemical identity, elemental analysis and MALDI-TOF mass spectrometry analysis in negative mode were used (see Table S2 and Figure S2). Both analyses verify the formation of the expected compound **1** with a clear peak at *m*/*z* = 797.8, corresponding to the [M^–^] molecular ion (see Figure S2).

Subsequently, building block **1** was subjected to a Suzuki–Miyaura cross-coupling reaction with two different diboronic compounds (Scheme 1): 1,4-phenyldiboronic acid and 4,7-bis­(4,4,5,5-tetramethyl-1,3,2-dioxaborolan-2-yl)-2,1,3-benzothiadiazole, leading to the formation of PAF-Phen (Schemes 1 and S2) and PAF-Phen-BT (Schemes 1 and S3) materials, respectively. The resulting materials were afforded as red thin powders in good yields after 72 h of reaction time (Table S1) and with elemental analysis in good agreement with theoretical values (Table S2).

**Scheme 1:**
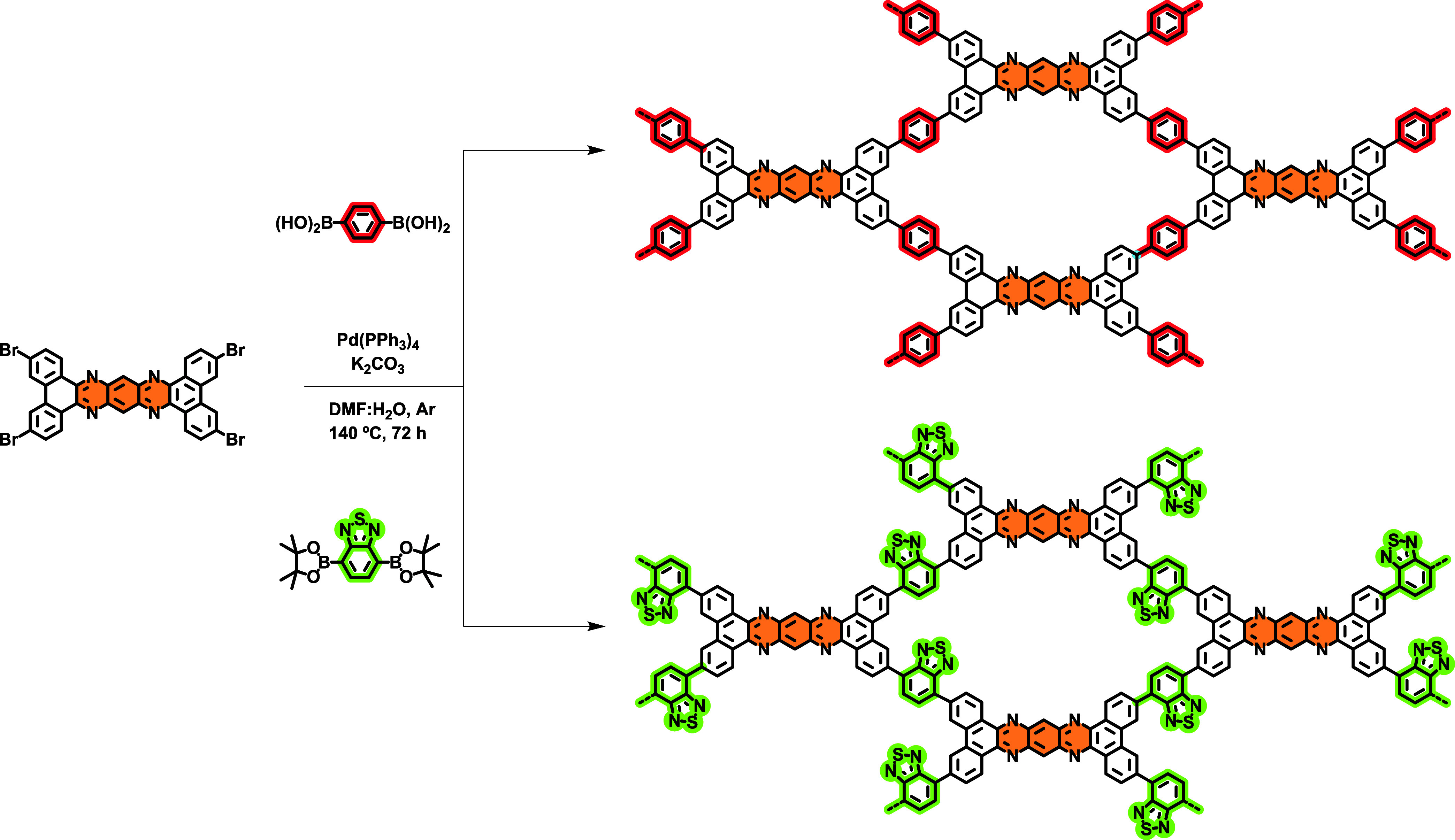
Schematic Representation of PAF-Phen (Red) and PAF-Phen-BT (Green)

Chemical characterization of each material was carried out by FTIR, solid-state CP/MAS ^13^C NMR, and XPS, confirming the successful formation of the materials. Comparative FTIR spectra between the PAF-Phen and PAF-Phen-BT materials with building block **1** confirmed the incorporation of the bisphenazine fragment into the backbone of the PAFs since its characteristic signals at 1343 and 1028 cm^–1^ remained in the spectra of both materials (Figures 1A and S3). The C=C signal at 1590 cm^–1^ from building unit **1** presented a shift to 1602 cm^–1^ in both materials. Furthermore, although residual terminal C–Br were still detectable in both PAF materials, the C–Br stretching vibrations of **1**, centered at 551 cm^–1^, largely diminished in the spectra of both materials, confirming the successful formation of PAF-Phen and PAF-Phen-BT by cross-coupling reaction.

**Figure 1:**
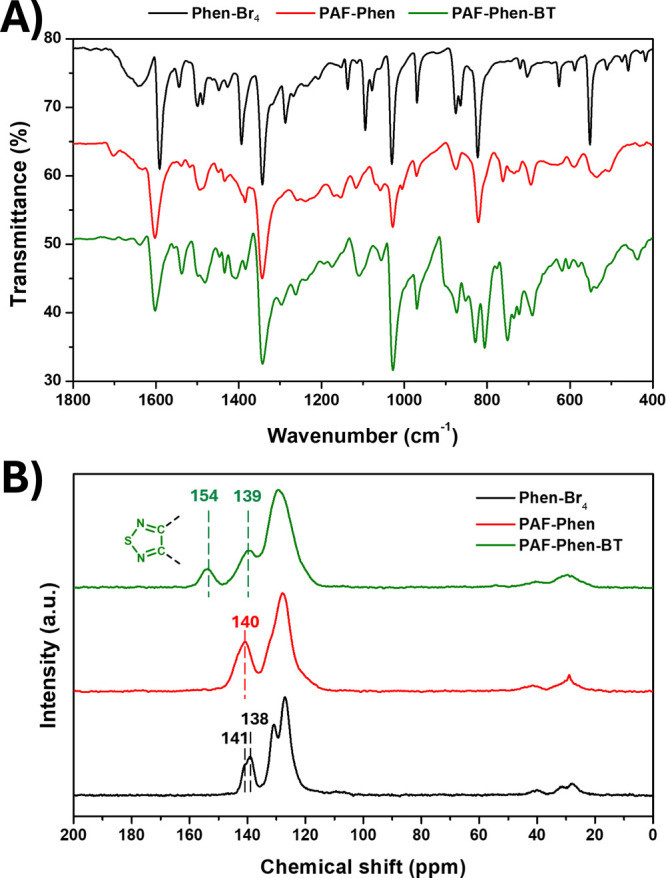
(A) FTIR spectra of building block Phen-Br_4_ (black), PAF-Phen (red), and PAF-Phen-BT (green). (B) Solid-state CP/MAS ^13^C NMR of the building block Phen-Br_4_ (black), PAF-Phen (red), and PAF-Phen-BT (green).

The solid-state CP/MAS ^13^C NMR spectra helped to enlighten the structural differences between these two materials. All samples present a main peak at 120–135 ppm, which corresponds to the C­(sp^2^) in the aromatic C=C bonds of the frameworks (Figure 1B). The C­(sp^2^) of the aromatic C=N bond from the bisphenazine fragment showed intense peaks at 140 ppm in the case of PAF-Phen, and 139 ppm for PAF-Phen-BT, which agree with the signal observed at 138 ppm (and the shoulder at 141 ppm) for building block **1**. Interestingly, PAF-Phen-BT shows an extra peak at 154 ppm, which is attributed to the −C=N– bond from the benzodiathiazole moiety present in its structure.

X-ray photoelectron spectroscopy (XPS) analysis provided additional insight into the chemical environment of both PAF materials (Figure 2). The survey XPS spectra (Figure S4A) confirms the presence of C and N atoms in both materials, in addition to S in PAF-Phen-BT. O and F atoms also appear in the survey spectra as contamination. The spectrum of the C 1s region (Figure 2A) shows an intense band centered at 285.3 eV that is slightly broader in the PAF-Phen-BT material. Fit analysis of the C 1s region displayed two main contributions at 285.3 and 286.3 eV (Figure 2B,C), which correspond to C­(sp^2^)-C­(sp^2^) and C­(sp^2^)-N­(sp^2^) bonds. The contribution of the latter signal is higher in the PAF-Phen-BT material compared to PAF-Phen, which is attributed to the presence of extra C=N bonds in the benzodiathiazole moiety.

**Figure 2:**
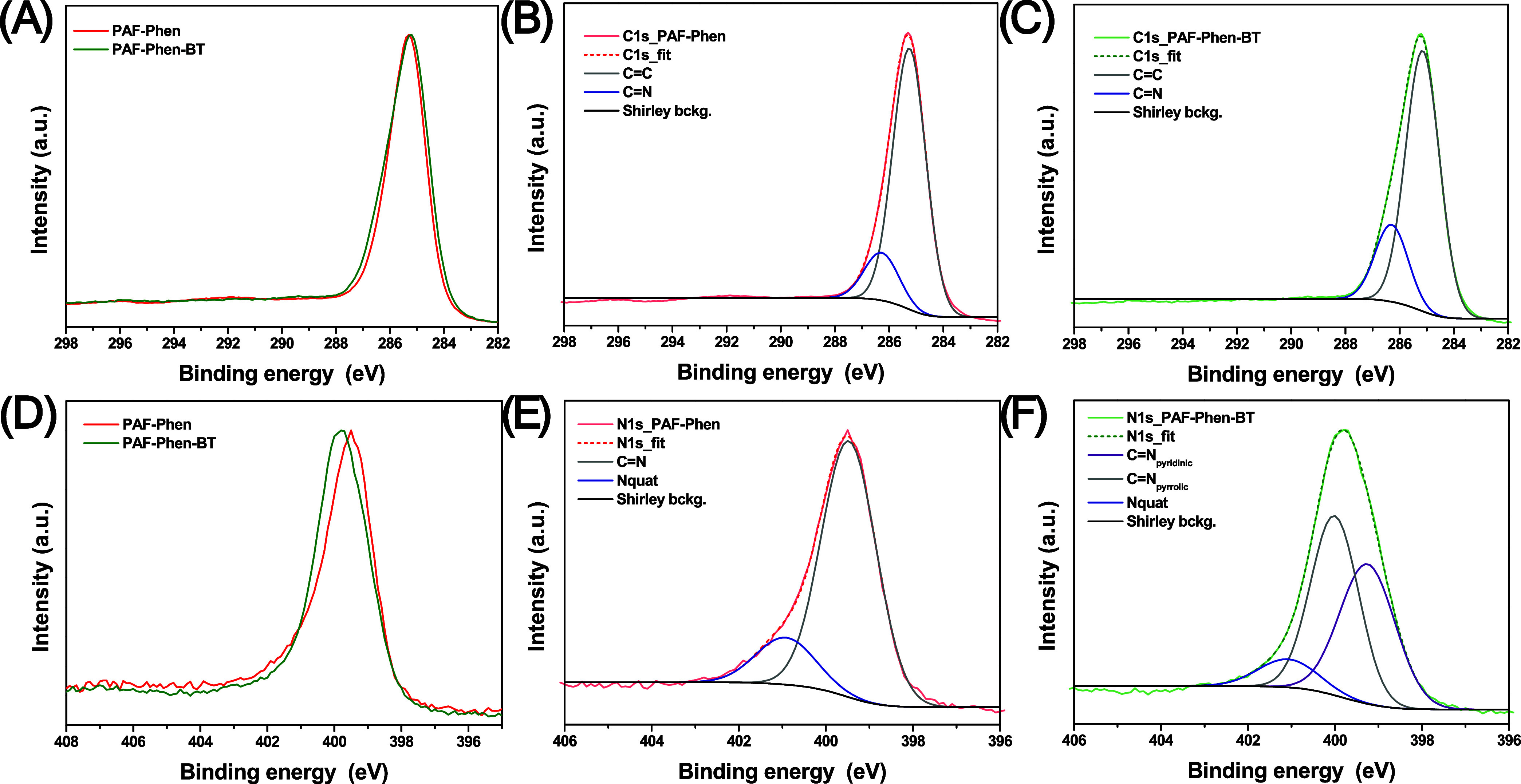
XPS spectra of (A) C 1s region of PAF-Phen (red) and PAF-Phen-BT (green) and corresponding fit peaks of (B) C 1s spectra of PAF-Phen and (C) C 1s spectra of PAF-Phen-BT. (D) N 1s region of PAF-Phen (red) and PAF-Phen-BT (green), and corresponding fit peaks of (E) N 1s spectra of PAF-Phen and (F) N 1s spectra of PAF-Phen-BT.

Additionally, spectra of the N 1s region (Figure 2D) show a broader band contribution in the PAF-Phen-BT material with the maximum shifted toward lower binding energies. Fit spectra of this region for PAF-Phen (Figure 2E) show a main contribution at 399.5 eV, corresponding to N­(sp^2^) atoms in the phenazine fragments and a minor component at 400.9 eV that indicates the presence of quaternary N atoms. For PAF-Phen-BT, the corresponding fit spectra (Figure 2F) present three contributions at 399.3 eV ascribed to the pyridinic N in phenazine fragments, at 400 eV ascribed to pyrrolic N in benzodiathiazole fragments, and a small contribution of oxidized N at 401 eV. The low proportion between quaternary and aromatic nitrogen confirms the high stability of the nitrogen-containing motifs in the frameworks. The presence of benzodiathiazole was also confirmed in the spectra obtained for S 2p (see Figure S4B), with bands centered at 165.9 and 167.1 eV that correspond to S 2p_3/2_ and S 2p_1/2_, respectively.

Diffuse reflectance UV–visible spectra of PAF-Phen and PAF-Phen-BT materials revealed photophysical absorption properties with broad absorption in the visible range for both materials (Figure 3A), although the onset wavelengths are red-shifted from PAF-Phen to PAF-Phen-BT material. Indirect optical band gaps were calculated by applying the Kubelka–Munk transformation to the absorption data (Figure S5). In particular, the band gap energies of PAF-Phen and PAF-Phen-BT were 1.91 and 1.70 eV, respectively. These results confirm that the presence of a benzothiadiazole linker instead of a phenyl ring leads to an increased and red-shifted absorption in the visible range. These observations are consistent with the properties of benzothiadiazole, a well-known electron-acceptor behavior, that has been reported to promote efficient charge separation and enable band gap tuning in donor–acceptor photoactive organic semiconductors.^26,27^

**Figure 3:**
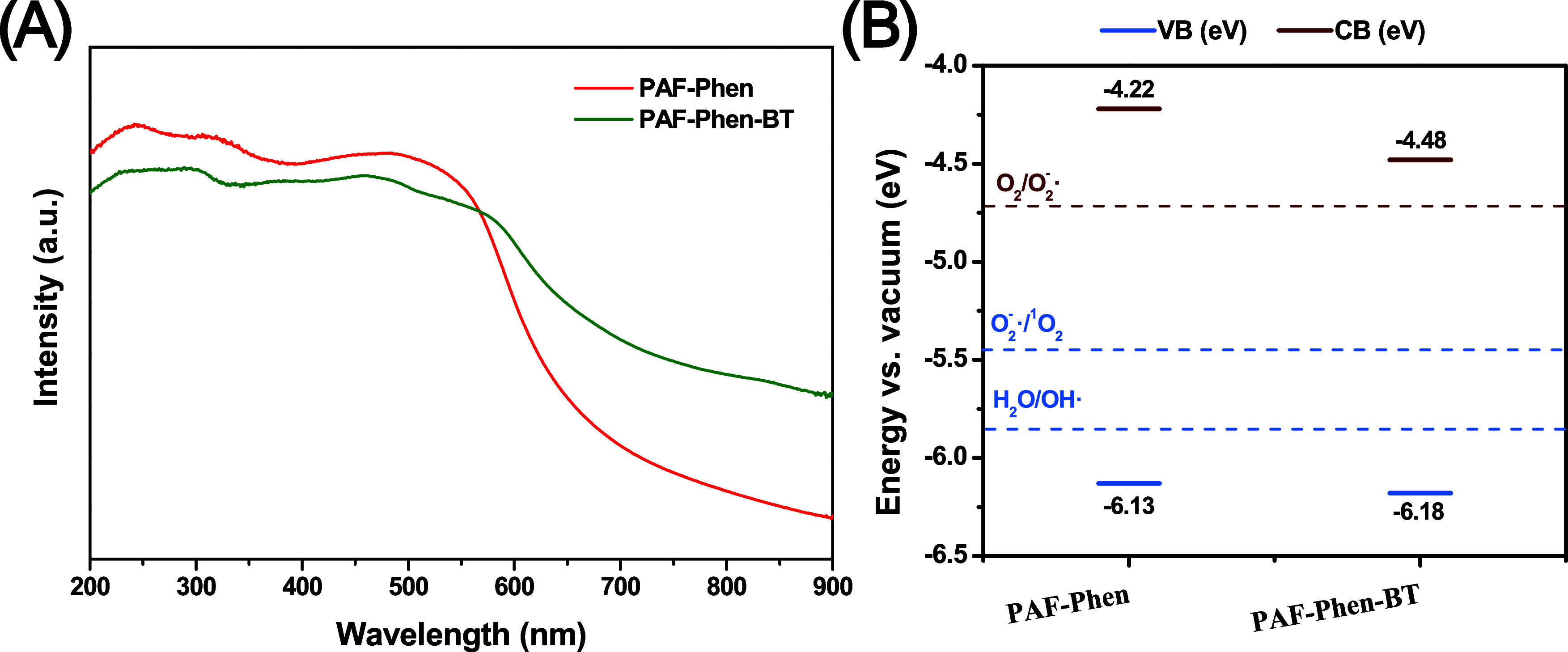
(A) Diffuse reflectance UV–visible spectra of PAF-Phen (red) and PAF-Phen-BT (green). (B) Energy diagram containing the valence band (VB) and conduction band (CB) energy position for PAF-Phen and PAF-Phen-BT materials estimated from the onset of the valence band XPS region and the optical band gap values. Relevant redox couples of ROS species have been included with dashed lines at their redox potential under the experimental conditions.

The analysis of the low binding energy region of the XPS spectra (0–25 eV range) for the PAFs allows us to determine the energy position of the valence band (Figures 3B and S6). Both materials present a similar valence band edge, with estimated energy values of 6.13 and 6.18 eV vs vacuum for PAF-Phen and PAF-Phen-BT materials, respectively. The obtained valence band energy values together with previously estimated optical band gap values allowed the construction of a band diagram for both materials (Figure 3B), which evidence their significant differences in the conduction band energy position. In particular, the conduction band energy of PAF-Phen is −4.22 eV vs vacuum while that for PAF-Phen-BT is −4.48 eV. Thus, the presence of an electron-acceptor moiety such as benzothiadiazole decreases the band gap and downshifts the conduction band position with respect to the corresponding material with phenyl units.

Scanning electron microscopy (SEM) images (Figure 4A,B) showed the laminar structure of both materials, consistent with the stacking of 2D layers. Transmission electron microscopy (TEM) observations further confirmed the layered nanostructured nature of PAF-Phen and PAF-Phen-BT (Figure 4C,D) and presented no evidence of crystallinity in the FFT image. Small differences were observed among the materials in terms of the particle size and roughness, with PAF-Phen-BT formed by smaller grains and having a rougher surface. X-ray diffraction patterns exhibit only broad, low-intensity features at high angles while lacking the characteristic intense low-angle reflections of crystalline porous frameworks (Figure S7), indicating the absence of a crystalline phase. These data are consistent with the TEM observations and the irreversible nature of the cross-coupling reaction that prevents self-healing of structural defects, necessary for its crystallization.^28^ Besides the amorphous nature of these materials, N_2_ adsorption isotherms have revealed moderate porosity for both materials (Figure S8A). In particular, the BET surface areas (total pore volume) have resulted in 144 m^2^/g (0.115 cm^3^/g) and 73 m^2^/g (0.074 cm^3^/g) for PAF-Phen and PAF-Phen-BT, respectively, evidencing a higher porosity of the PAF-Phen material, relative to PAF-Phen-BT. However, both materials present a similar pore size distribution with pores ranging from 1 to 8 nm (Figure S8B).

**Figure 4:**
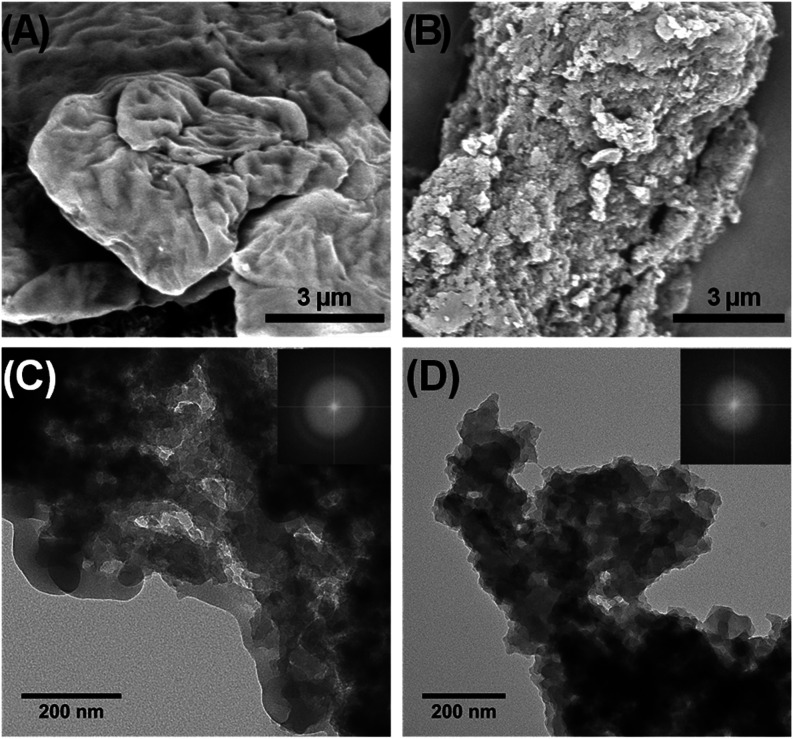
SEM images of (A) PAF-Phen and (B) PAF-Phen-BT and TEM images with corresponding FFT images in the inset of (C) PAF-Phen and (D) PAF-Phen-BT.

In addition, the thermal stability of both materials was evaluated using thermogravimetric analysis (TGA) under air atmosphere. Both materials exhibit good thermal stability and keep their structural integrity up to 529 and 560 °C for PAF-Phen and PAF-Phen-BT, respectively (Figure S9).

### Photocatalytic Study

To evaluate the photocatalytic efficiency of PAF-Phen and PAF-Phen-BT, we first assessed their performance in degradation of the prototypical azo dye para red (PR). The reaction progress was monitored by the decrease of its characteristic absorption band in the visible region at λ ≈ 485 nm (see Experimental Section). This dye is widely recognized as a model water pollutant, commonly released into aquatic environments by textile and printing industries.^8^ Notably, PAF-Phen exhibited superior photocatalytic activity compared to its parent material, PAF-Phen-BT (Figure S10). While PAF-Phen achieved nearly complete degradation of para red within 180 min, PAF-Phen-BT exhibited negligible catalytic activity under identical conditions, showing a degradation rate comparable to the control photolysis experiment (irradiation without catalyst). An overview of the available literature (Table S3) indicates that the photocatalytic activity of PAF-Phen is comparable to that of state-of-the-art photocatalytic organic materials for azo dyes degradation.

Kinetic studies indicated a zero-order photocatalytic reaction (Figure 5A), with a kinetic constant of 4.73·10^–3^ min^–1^ for PAF-Phen and 4.33·10^–4^ min^–1^ for PAF-Phen-BT. The kinetics of para red degradation showed a behavior independent of the initial dye concentration, suggesting that the rate-determining step of this process is likely the generation of reactive oxygen species. The involvement of molecular oxygen as the terminal oxidant in the catalytic cycle was confirmed by the null conversion observed when the reaction was conducted under an argon atmosphere (Figures 5B and S11). Therefore, photocatalytically generated reactive oxygen species are likely the ultimate agents responsible for para red degradation.

**Figure 5:**
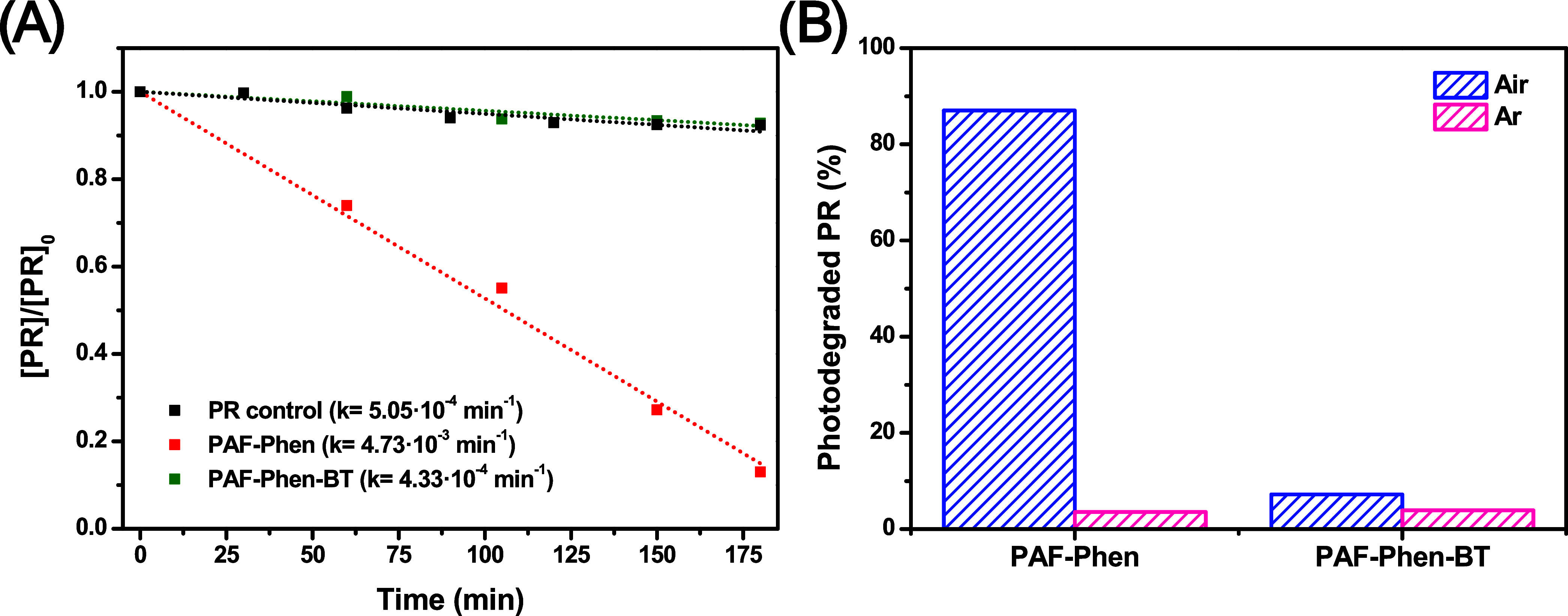
(A) Zero-order kinetic traces ([PR]/[PR]_0_, where [PR] corresponds to PR concentration at a given time and [PR]_0_ corresponds to initial PR concentration) for the photodegradation of para red without catalysts (black) and using PAF-Phen (red) and PAF-Phen-BT (green) catalytic materials under irradiation with blue light for 3 h. (B) Bar diagram with the photodegradation results of PR [(1-[PR]/[PR]_0_)*100] using both materials under air (blue) and Ar (pink) atmospheres.

To elucidate the nature of reactive oxygen species involved in para red degradation, we first performed the photocatalytic reaction using PAF-Phen as the catalyst in the presence of 1,4 diazabicyclo[2.2.2]­octane (DABCO), a selective quencher for singlet oxygen (^1^O_2_).^29^ Remarkably, DABCO completely suppressed the oxidative degradation of para red (Figure S12B), indicating that singlet oxygen is the primary oxidizing species responsible for this transformation. Singlet oxygen generation is typically explained by energy-transfer processes that do not involve electron transfer between the photocatalyst and molecular oxygen.^29^ However, an alternative pathway has been reported in which electron transfer produces superoxide radical anions (O_2_^•-^) that can subsequently yield singlet oxygen through a secondary reduction process.^30^ To assess the relevance of this indirect route, we tested benzoquinone, a specific quencher for superoxide anions.^31^ Interestingly, para red degradation continued even with benzoquinone present, though at lower efficiency, with about 60% of the catalytic activity preserved (Figure S12C,D). These observations suggest that para red is primarily oxidized by singlet oxygen generated via an energy-transfer mechanism. Nevertheless, the 40% inhibition observed with benzoquinone indicates that an electron-transfer-mediated pathway involving a superoxide intermediate cannot be excluded. This secondary mechanism could account for up to 40% of PR degradation.

We subsequently investigated the photocatalytic activity of these materials in the photooxidation of furfuryl alcohol (FA). ^1^H NMR analysis of the reaction mixtures confirmed that the reaction proceeds selectively toward the oxidation of FA to 5-hydroxy-2­(5H)-furanone (5H5F) (Scheme 2). This four-carbon lactone is of practical importance as a key intermediate in the synthesis of valuable chemicals, including maleic acid, 1,4-butanediol, and γ-butyrolactone.^32^ In this case, PAF-Phen also exhibited superior photocatalytic efficiency (100% conversion) compared to PAF-Phen-BT (22% conversion). Null conversion was observed in the control experiment under dark conditions using the most active photocatalyst. Under light irradiation of PAF-Phen, conversion of FA to 5H5F was significantly faster than para red degradation, with complete conversion achieved in less than 90 min (see experimental details in the Supporting Information and Figures S13–S15).

**Scheme 2:**
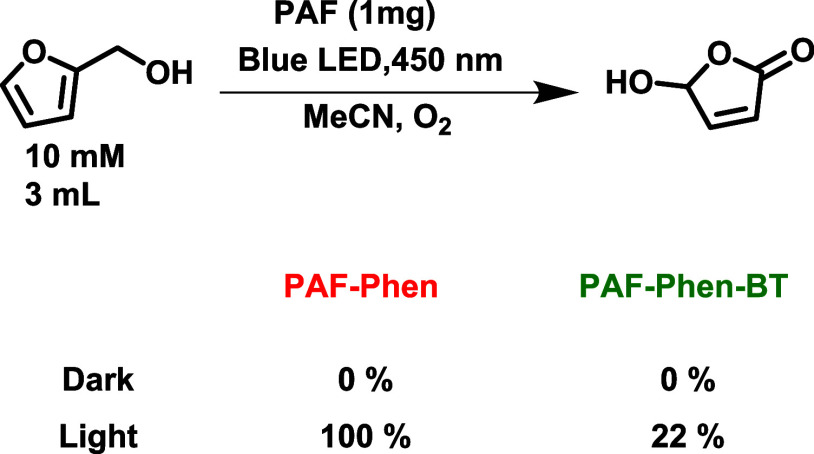
Photooxidation Experiments of Furfuryl Alcohol with PAF-Phen and PAF-Phen-BT^a^

a The reported percentage values refer to the conversions measured by ^1^H-NMR peak integration from the crudes.

FA oxidation was completely quenched by the addition of DABCO (Figure S16), whereas benzoquinone did not significantly suppress the formation of 5H5F. These results suggest that, under the reaction conditions, quantitative oxidation of FA was achieved via ^1^O_2_ generation following an energy-transfer process. Indeed, reducing the overall singlet oxygen production by suppressing the electron-transfer pathway (in the presence of benzoquinone) is not sufficient to affect the rapid oxidation of FA. Overall, the data suggest that singlet oxygen formation occurs mainly via energy transfer, with electron-transfer-based generation remaining possible but secondary in the photocatalytic system of FA oxidation, which accounts for approximately 15% of the catalytic conversion (see Scheme 3).

**Scheme 3:**
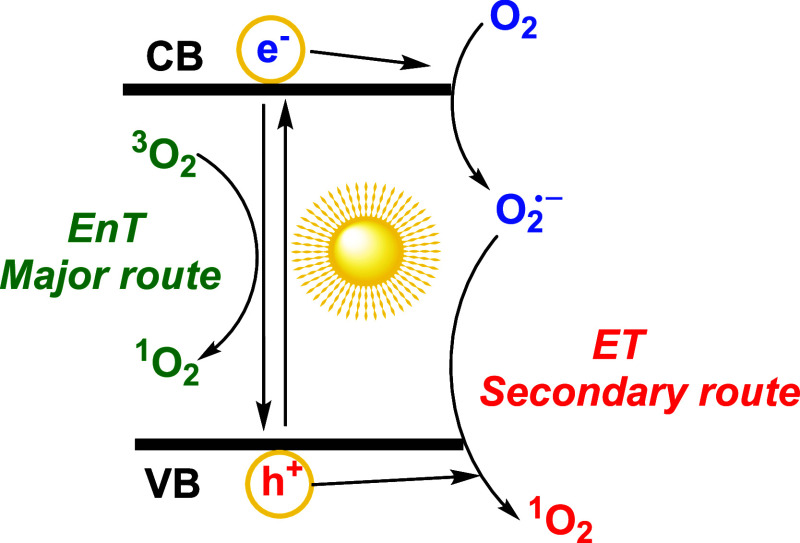
Schematic representation of Singlet Oxygen Generation by Irradiation of Semiconductor Materials That Can be Formed through Energy-Transfer Mechanisms (Green) from ^3^O_2_ or through Electron-Transfer Mechanisms (Blue and Red) Generating First Superoxide Anion Radical That Later Evolves to Singlet Oxygen Species

The practical utility of the most active photocatalyst, PAF-Phen, relies on its ability to be reused across multiple catalytic cycles. To evaluate this, we examined its recyclability using the photooxidation of furfuryl alcohol as a model reaction. Notably, the material retained its photocatalytic performance for up to five consecutive runs without significant loss of activity (Figure S17). Hot-filtration experiments confirmed the absence of catalyst leaching (Figure S18), supporting the strictly heterogeneous nature of the photocatalytic process. Importantly, FTIR and XPS analyses of the recovered PAF-Phen photocatalyst confirmed preservation of the chemical integrity of the material after photocatalytic conditions (Figures S19 and S20).

### Electrocatalytic Study

As an initial assessment of the electrochemical behavior of PAF-Phen and PAF-Phen-BT, cyclic voltammetry was performed on glassy carbon (GC) electrodes modified with each material, both in the absence and presence of O_2_ (Figure 6A). The lack of electrochemical peaks under oxygen-free conditions confirms the absence of electroactive groups in both materials. Subsequently, the resistance to electron transfer was evaluated using electrochemical impedance spectroscopy (EIS). Figure 7A shows the Nyquist plots obtained from EIS experiments employing the ferro-/ferricyanide couple as a redox probe. Interestingly, PAF-Phen/GC exhibits the lowest electron-transfer resistance compared to PAF-Phen-BT/GC and the GC reference electrode. The null activity under an Ar atmosphere and the lower charge-transfer resistance in PAF-Phen/GC are consistent with the superior photocatalytic activity of PAF-Phen, which relies on more efficient electron-transfer pathways than PAF-Phen-BT.

**Figure 6:**
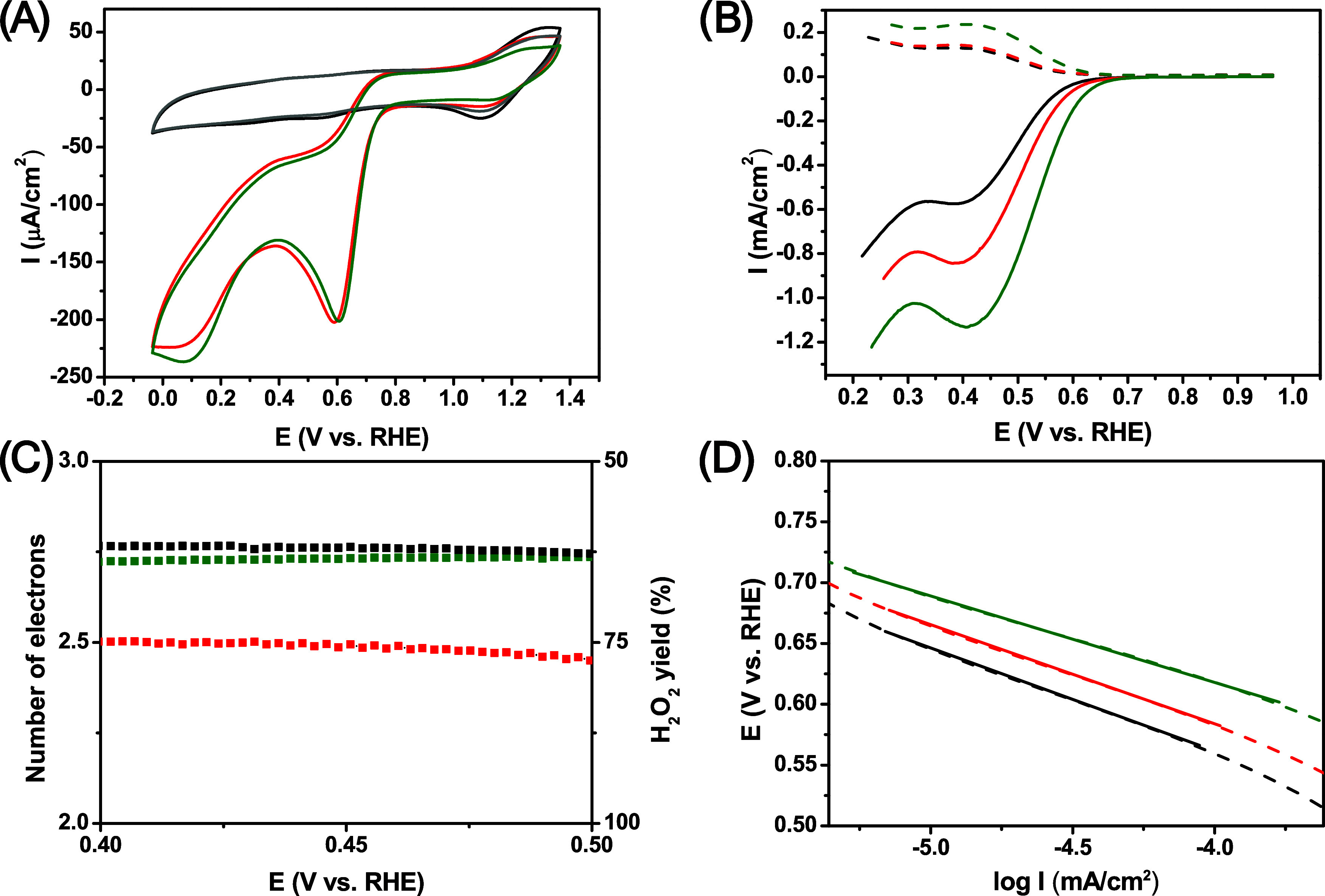
(A) Cyclic voltammograms of PAF-Phen/GC (red) and PAF-Phen-BT/GC (green) in the presence of oxygen and PAF-Phen/GC (black) and PAF-Phen-BT/GC (gray) in the absence of oxygen in 0.1 M KOH, pH 13. (B) Hydrodynamic linear sweep voltammetry of GC (black), PAF-Phen/GC (red), and PAF-Phen-BT/GC (green) in O_2_-saturated 0.1 M KOH solution at 1600 rpm and 10 mV/s. Continuous line disk current and discontinuous line ring current. (C) Number of electrons calculated for ORR using RRDE at different potentials and electrochemical yield of H_2_O_2_ in percentage obtained from RRDE data. (D) Tafel slopes.

**Figure 7:**
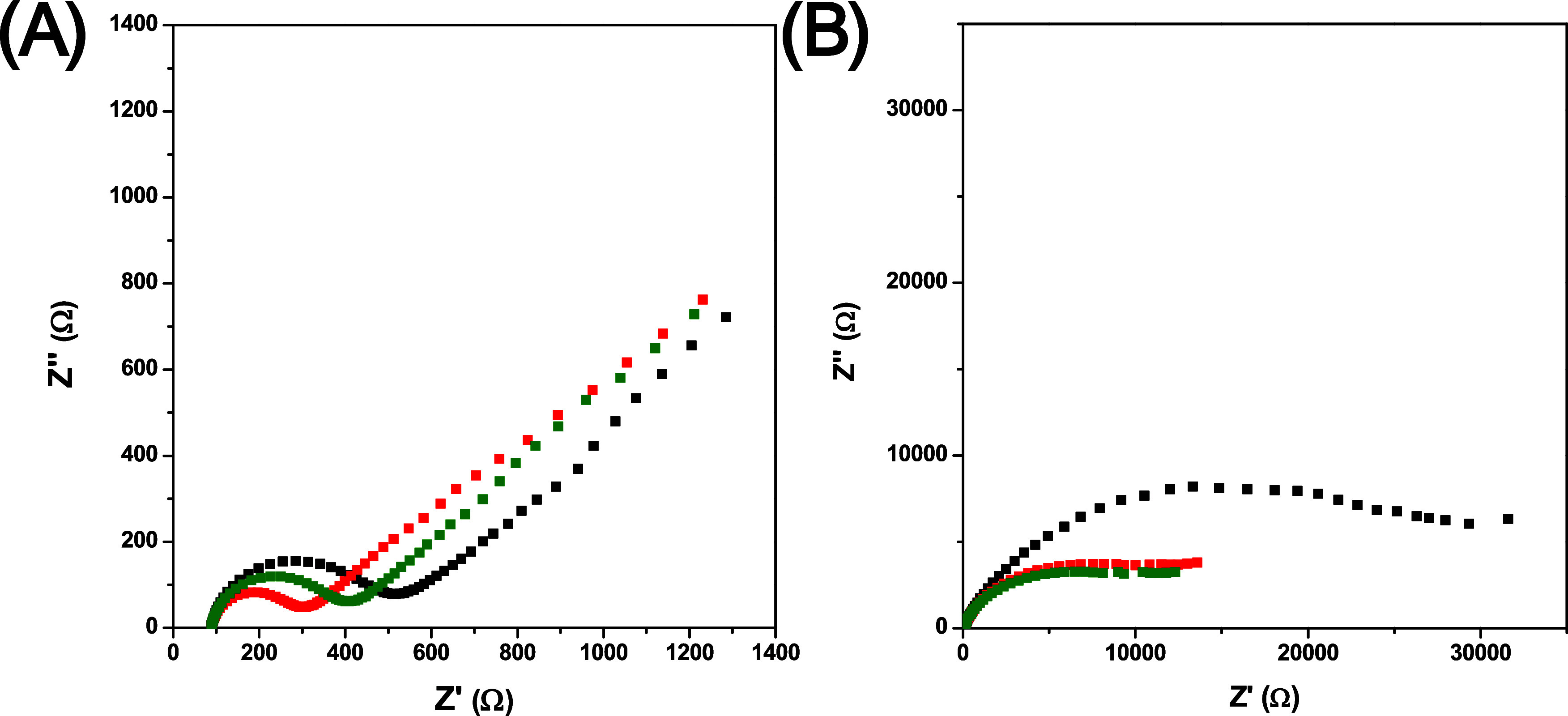
(A) Nyquist plots recorded in 0.1 M KCl containing 1.0 × 10^–2^ M ferrocyanide/ferricyanide system at a GC (black), PAF-Phen/GC (red), and PAF-Phen-BT/GC (green). (B) Nyquist plots recorded in 0.1 M KOH, pH 13, saturated with oxygen.

The electrocatalytic role of PAF-Phen and PAF-Phen-BT in the oxygen reduction reaction (ORR) was evaluated under hydrodynamic conditions by using a modified Pt/GC ring disk RRDE setup in an oxygen-saturated atmosphere. As deduced from hydrodynamic linear sweep voltammetry (HLSV) measurements (Figure 6B) and summarized in Table 1, the ORR onset potential improved by 22 mV for PAF-Phen/GC and 49 mV for PAF-Phen-BT/GC compared with that of bare GC. A notable difference in current intensity was also observed, with PAF-Phen-BT/GC exhibiting the highest increase, followed by PAF-Phen/GC. Therefore, PAF-Phen-BT proves to be the most efficient electrocatalyst for the ORR, while PAF-Phen demonstrates the highest photocatalytic activity for the PR degradation and FA oxidation.

**Table 1:** Summary of the Main Electrochemical Parameters for ORR of GC, PAF-Phen/GC, and PAF-Phen-BT/GC

electrode	*E*_onset_ vs RHE (V)	i lim (mA/cm^2^)	Tafel slope (mV/dec)	number of electrons	% H_2_O_2_
GC	0.585	–0.576	87.8 (*n* = 1.35)	2.50	75
PAF-Phen/GC	0.607	–0.844	81.7 (*n* = 1.44)	2.75	62
PAF-Phen-BT/GC	0.634	–1.213	70.4 (*n* = 1.67)	2.70	63

To evaluate the contribution of the electroactive area of the modified electrodes, their electrochemical surface area (ECSA) was determined via cyclic voltammetry (Figure S21) by measuring the capacitive current in the nonfaradaic region under oxygen reduction reaction conditions. Interestingly, while the PAF-Phen-BT material exhibits a lower BET specific surface area compared to PAF-Phen (72.6 vs 143.7 m^2^/g), its ECSA is significantly larger (4.17 vs 3.15 cm^2^). To decouple the effects of electrochemical surface area from intrinsic activity, the linear sweep voltammetry curves under ORR conditions were normalized by the ECSA of each electrode (Figure S22), contrasting with the geometric area normalization used in Figure 6B. As shown, the ECSA-normalized ORR current density for PAF-Phen-BT remains markedly higher. Furthermore, PAF-Phen-BT displays a less negative onset potential, indicating accelerated electrochemical kinetics, particularly during the O_2_ adsorption steps, which is well-supported by our density functional theory calculations (*vide infra*). Collectively, these findings demonstrate that the electrocatalytic performance is not merely a function of the total available surface area but is dictated by the intrinsic quality and efficiency of the active sites. The previously reported data summarized in Table S4 suggest that the ORR performance of our materials compares well to that of other metal- and pyrolysis-free organic polymer electrocatalysts reported in the literature.

Oxygen reduction can proceed via a four-electron pathway, ultimately generating water, or through a two-electron mechanism that produces H_2_O_2_.^33^ To distinguish between these two processes, we electrochemically monitored the formation of H_2_O_2_ by measuring the current at the platinum ring electrode, which corresponds to the oxidation of H_2_O_2_ and increases as ORR progresses. The largest increase was observed for PAF-Phen-BT/GC, indicating higher H_2_O_2_ production. Since H_2_O_2_ formation is directly linked to the number of electrons involved in ORR (Figure 6C), a greater amount of H_2_O_2_ suggests a mechanism closer to the two-electron pathway. By comparing the current at the GC-modified disk electrodes with that at the platinum ring, we determined that PAF-Phen/GC and PAF-Phen-BT/GC proceed via 2.75 and 2.70 electrons, respectively (Figure 6C). This result indicates a mixed 2 + 2 mechanism, with a predominant contribution from the two-electron pathway rather than four-electron reduction to water.^34^

The Tafel slope data (Table 1 and Figure 6D) provide valuable insights into the ORR mechanism for each material. A lower Tafel slope indicates more efficient electrocatalytic performance, whereas a higher slope reflects greater polarization and thus, less favorable catalytic activity.^35^ A smaller Tafel slope also corresponds to a lower overpotential for ORR.^36^ In a conventional Tafel analysis, plotting log­(*i*) versus overpotential (η) assuming a charge-transfer coefficient (α) of 0.5 allows estimation of the number of electrons involved in the rate-determining step (RDS).^37^ According to the relationship Tafel slope = 0.059/(α·n), a slope of 118 mV·dec^–1^ corresponds to *n* = 1, while a slope of 59 mV·dec^–1^ corresponds to *n* = 2.^38^ The bare GC electrode exhibits a Tafel slope of 87.8 mV·dec^–1^, while PAF-Phen/GC shows a slope of 81.7 mV·dec^–1^, corresponding to electron numbers of 1.35 and 1.44, respectively. In contrast, PAF-Phen-BT/GC presents a Tafel slope of 70.4 mV·dec^–1^, which corresponds to *n* ≈ 1.67. These results suggest that while PAF-Phen operates through a mechanism where the first electron transfer is likely the rate-determining step, for PAF-Phen-BT, the first electron transfer is more favored and therefore appears less critical to the overall electrochemical kinetics.

The slow first electron transfer for PAF-Phen/GC can be demonstrated by EIS analysis under ORR conditions (E_onset potential and oxygen saturation) (Figure 7B). In contrast to the behavior observed in the absence of oxygen using the ferro/ferricyanide couple as a redox probe, under ORR conditions, PAF-Phen-BT/GC favors charge-transfer processes. All experimental evidence suggests that PAF-Phen-BT performs better as an electron-transfer mediator only in the presence of oxygen, whereas PAF-Phen acts as a superior photocatalyst and shows lower resistance to electron transfer under oxygen-free conditions. This shift in catalytic behavior is attributed to an enhanced interaction with molecular oxygen, which increases its surface concentration and favors the reduction of O_2_.^39^

### Theoretical Calculations

To elucidate the mechanisms underlying the different photo- and electrocatalytic behaviors of PAF-Phen and PAF-Phen-BT, we carried out theoretical calculations within the density functional theory (DFT) framework. The materials were represented using tetrameric cluster models, as shown in Figure 8 (see Figures S23 and S24 and also Section S16). Although these models do not include long-range periodicity, they capture the local electronic effects associated with linker substitution, which is the main focus of the present comparative study.

#### Electrocatalysis

We computed the vertical ionization (VIE) and electron attachment energies (EAEs) of PAF-Phen and PAF-Phen-BT models (Table 2). These descriptors reflect, respectively, the ability of the neutral species to release an electron and the radical cation to subsequently capture it. The computed VIE values are consistent with the valence band energies obtained from XPS measurements (Figure 3). Given the small differences between both systems, especially within the expected DFT error, we infer that the superior electrocatalytic performance of PAF-Phen-BT in the ORR is not primarily related to its intrinsic electron-donating ability but rather to dynamic interfacial processes, such as O_2_ adsorption on the surface.

**Table 2:** Vertical Ionization and Electron Attachment Energies of PAF-Phen and PAF-Phen-BT

	VIE (eV)	EAE (eV)
PAF-Phen	6.17	6.16
PAF-Phen-BT	6.16	6.12

In this context, we evaluated the adsorption energies of a single O_2_ molecule on different optimized adsorption sites of the model tetramer in vacuum. These positions, shown in Figure 8, are (**1**) within the pore in a confined region near the linker junction, (**2**,**3**) on top of the linker unit, in π-stacked configurations, and (**4**) on top of the phenazine core, in a π-stacked configuration. For each site, an O_2_ molecule was positioned in a plausible initial configuration, and the resulting structure was subsequently optimized at the DFT level. These calculations provide a qualitative assessment of the O_2_–catalyst interaction, which is regarded as one of the primary factors underlying the differences observed in the electrocatalytic performance.

**Figure 8:**
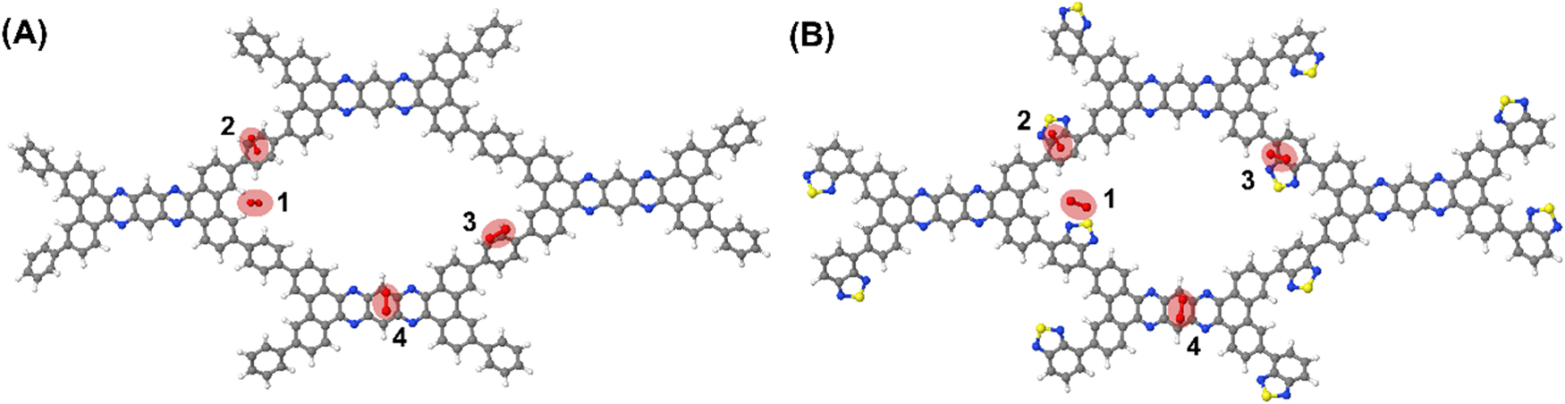
Structures of (A) PAF-Phen and (B) PAF-Phen-BT tetramers, indicating the adsorption positions of one O_2_ molecule.

The adsorption energies are superior in the pore region for both systems, which is the only configuration where oxygen is not stabilized by π-stacking interactions with the aromatic rings. Notably, the adsorption energy in the pore of PAF-Phen-BT is 34% stronger than that of PAF-Phen (Table 3), likely due to enhanced polarization (induction) effects arising from the heterogeneous electron density of the benzothiadiazole units in this region. O_2_ also interacts more strongly with the BT linkers than with the phenyl linkers, either in parallel (17%) or perpendicular (20%) orientations, while, as expected, the interaction at the bisphenazine region, common to both materials, is almost identical (0.2%).

**Table 3:** Adsorption Energies of O_2_ on the Positions of PAF-Phen and PAF-Phen-BT Tetramers Shown in Figure 8

	*E*_ads_ PAF-Phen (kcal/mol)	*E*_ads_ PAF-Phen-BT (kcal/mol)
**1**	–1.72	–2.31
**2**	–1.58	–1.90
**3**	–1.63	–1.90
**4**	–1.45	–1.46

Overall, the superior electrocatalytic activity of PAF-Phen-BT can be attributed to a more favorable interaction with O_2_ molecules. The enhanced O_2_ affinity is consistent with the improved ORR activity observed experimentally, including a less negative onset potential and a higher current density. Therefore, the combined experimental and theoretical evidence suggests that the stronger O_2_–catalyst interaction in PAF-Phen-BT facilitates the ORR process, providing a coherent mechanistic rationale for its superior electrocatalytic performance.

#### Photocatalysis

We attribute the superior photocatalytic activity of PAF-Phen compared with that of PAF-Phen-BT in the presence of oxygen to the photosensitization of molecular oxygen, leading to singlet oxygen formation. To clarify the difference between the two materials, we modeled their light absorption and subsequent deactivation pathways.

The computed absorption spectra are consistent with the experimental diffuse reflectance spectra (Figure S25), showing a red shift of the first absorption band of PAF-Phen-BT relative to PAF-Phen (2.87 vs 3.02 eV). The calculations are not intended to reproduce the full experimental absorption spectra, since they correspond to a single optimized tetramer and include only the lowest excited states. Accordingly, the additional experimental absorption features likely arise from the heterogeneity of the amorphous frameworks, higher-energy electronic transitions, and vibronic broadening. In the model clusters, the first absorption band is ascribed to the population of the first two excited singlet states of each molecule (S_1_ and S_2_), which are degenerate. The associated transitions are of π→π* character in both systems, with both π and π* orbitals delocalized over several bisphenazine units and their linkers (Figure S26).

Relaxation from these states leads to S_1_ minima at 2.73 eV for PAF-Phen and 2.35 eV for PAF-Phen-BT (Figure 9), relative to their respective Franck–Condon ground states. To assess the photosensitizing abilities, we computed the spin–orbit coupling matrix elements of the lowest 30 triplet states with S_1_, H^SO^(S_1_–T_n_) (Table S5). The key difference between both systems lies in the accessibility of the intersystem crossing (ISC) to the triplet manifold. In PAF-Phen, the S_1_ minimum is nearly degenerate with a triplet state, T_11_ (0.07 eV energy difference), with a relatively large coupling value H^SO^(S_1_–T_11_) 8.12 cm^–1^. In contrast, for PAF-Phen-BT, the triplets degenerate with the S_1_ minimum showcoupling values H^SO^(S_1_–T_n_) below 1 cm^–1^. The largest coupling (1.42 cm^–1^) corresponds with T_21_, located 0.20 eV above S_1_. Although one might expect stronger spin–orbit coupling in PAF-Phen-BT, owing to the heavier sulfur atoms, the nonbonding orbitals of these sulfurs, which would enhance the S–T coupling according to El Sayed rules, are lower in energy and are not expected to be directly accessed from the S_1_ minima.

**Figure 9:**
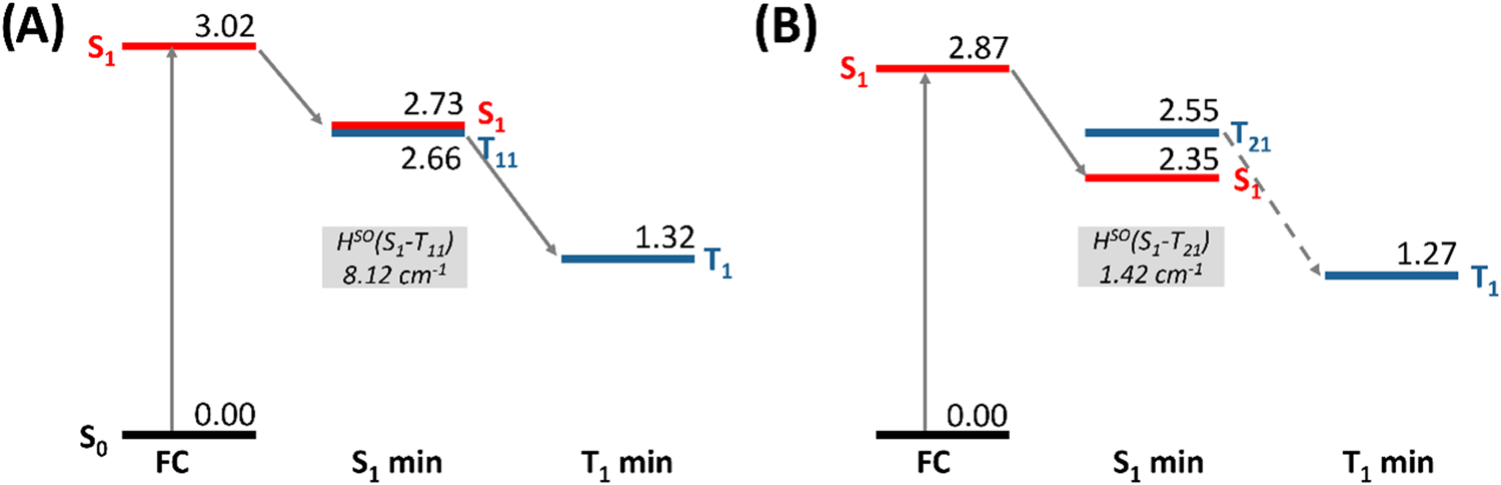
Deactivation pathways of (A) PAF-Phen and (B) PAF-Phen-BT. Energies in eV with respect to the Franck–Condon (FC) S_0_ state. The largest H^SO^(S_1_–T_n_) values at the S_1_ minima are included.

Relaxation of the corresponding triplets populated by ISC leads to T_1_ minima at 1.32 eV for PAF-Phen and 1.27 eV for PAF-Phen-BT. From both minima, triplet–triplet energy transfer to ^3^O_2_ to yield ^1^O_2_ is energetically feasible, as both energies exceed that of ^1^O_2_ generation (0.98 eV).^40^ Additionally, we computed the adiabatic energy of the lowest triplet state of PR and FA at 1.71 and 4.07 eV, respectively, at the same level of theory. Since these values lie above the T_1_ minima of both materials, direct triplet–triplet energy transfer to these molecules is unlikely to be a relevant photodegradation pathway. Overall, these results indicate that the enhanced photosensitized degradation of PR and FA by PAF-Phen arises from the more efficient formation of ^1^O_2_ driven by higher ISC rates compared with PAF-Phen-BT.

## Conclusions

In this work, two closely related bisphenazine-based porous aromatic frameworks, PAF-Phen and PAF-Phen-BT, were synthesized and investigated as metal-free photo- and electrocatalytic nanostructured materials for oxygen activation. Despite their similar structural motifs and optical properties, these frameworks exhibit markedly different catalytic behaviors, underscoring the decisive role of subtle electronic and structural modifications.

Incorporation of the benzothiadiazole linker in PAF-Phen-BT enhances electron-transfer processes under electrochemical conditions, resulting in improved oxygen reduction reaction performance. In contrast, PAF-Phen displays superior photocatalytic activity in aerobic oxidation reactions, operating predominantly through energy-transfer-mediated photosensitization of molecular oxygen to generate singlet oxygen.

Combined experimental and theoretical analyses indicate that the enhanced electrocatalytic performance of PAF-Phen-BT does not originate from a higher intrinsic electron-donating ability. Instead, PAF-Phen-BT shows significantly stronger adsorption of molecular oxygen, particularly within the pore regions and at the benzothiadiazole linkers, arising from enhanced polarization effects and heterogeneous electron density. This stronger O_2_ binding favors interfacial electron-transfer processes under electrochemical conditions, lowering charge-transfer resistance and promoting oxygen reduction.

Conversely, the superior photocatalytic activity of PAF-Phen originates from its excited-state properties. Time-dependent DFT calculations reveal a more favorable intersystem-crossing pathway in PAF-Phen, characterized by near-degeneracy between the S_1_ state and low-lying triplet states together with significantly larger spin–orbit coupling. These features enable efficient population of long-lived triplet states, facilitating effective energy transfer to molecular oxygen and promoting singlet oxygen generation. Although the triplet-state energies of both materials are sufficient to sensitize O_2_, the higher intersystem-crossing efficiency in PAF-Phen leads to superior photosensitization and photocatalytic performance.

Taken together, these results demonstrate that photocatalytic and electrocatalytic activities in bisphenazine-based porous frameworks are governed by distinct and nontransferable descriptors: excited-state dynamics and intersystem-crossing efficiency control energy-transfer-driven photocatalysis, whereas oxygen adsorption strength and interfacial polarization dominate electron-transfer-mediated electrocatalysis. This study therefore establishes bisphenazine-based porous aromatic frameworks as a model platform to disentangle EnT and ET processes and provides clear guidelines for the rational design of organic materials with tailored photo- or electrocatalytic functions, underscoring the importance of mechanistic specificity in the development of porous organic catalysts for photo- and electrochemical applications.

## Supplementary Material



## Notes

Additional experimental details, characterization (including NMR, FTIR, XPS, DRS, XRD, TGA, BET, and UV–vis spectroscopy), and electrochemical and theoretical details (PDF)
