# Supplementary material for: Bisphenazine-Based Porous Aromatic Frameworks for Photocatalytic and Electrocatalytic Oxygen Activation
Source: ACS Appl Nano Mater. 2026 Jul 29;9(31):14884–96. doi: 10.1021/acsanm.6c02207 (PMC13471794; doi:10.1021/acsanm.6c02207)
Supplement: Supplementary file 1 [file an6c02207_si_001.pdf]

SUPPORTING INFORMATION

Bisphenazine-Based Porous Aromatic Frameworks for Photocatalytic and Electrocatalytic Oxygen Activation

Miguel Sánchez-Fuente,[±] *Juan Manuel Hernández-Gómez,*[√] *Julia Arnanz,*[§] *Víctor Cepa-López,* [±] *Sonia Bruña,*[¥,±] *Emiliano Martínez-Periñán,* [¥,√] *Alicia Moya,* [¥,±,*] *Inés Corral,*[¥,§,*] *Cristina Gutiérrez-Sánchez,*[¥,√,*] *and Rubén Mas-Ballesté.* [¥, ±,*]

± Department of Inorganic Chemistry (Module 7), Facultad de Ciencias, Universidad Autónoma de Madrid, 28049 Madrid, Spain.

√ Department of Analytical Chemistry (Module 16), Facultad de Ciencias, Universidad Autónoma de Madrid, 28049 Madrid, Spain.

§ Department of Chemistry (Module 13), Facultad de Ciencias, Universidad Autónoma de Madrid, 28049 Madrid, Spain.

¥ Institute for Advanced Research in Chemical Sciences (IAdChem), Universidad Autónoma de Madrid, 28049 Madrid, Spain.

Email. ruben.mas@uam.es, cristina.gutierrezs@uam.es, ines.corral@uam.es, alicia.moya@uam.es

Contents

1. General Materials and Methods
2. Computational details
3. Synthesis of the building block Phen-Br_4 (**1**)
4. Synthesis of PAF materials
   4.1 Synthesis of PAF-Phen
   4.2 Synthesis of PAF-Phen-BT
5. Comparative FTIR spectra of PAF materials and their respective precursors
6. XPS analysis of the materials
7. Kubelka Munk calculations for the optical band gap of the materials
8. Analysis of the XPS valence band region
9. X-ray Diffraction of the materials
10. Thermogravimetric analysis of the materials
11. Nitrogen Isotherms of the materials
12. Photodegradation of para red (PR)
13. ^{1}H-NMR spectra of FA oxidation experiments
14. FTIR spectra after recyclability test
15. Computational models
16. References

1. General Materials and Methods

Materials and instrumentation: All the starting reagents and solvents were purchased from BLD Pharm, Sigma Aldrich, or TCI.

Thermogravimetry Analysis (TGA)

Thermogravimetry Analysis (TGA) was carried out with a Thermogravimetry/Differential Thermal Analysis (TG-DTA) analyzer within the temperature range of 25–800°C with a heating rate of 10 °C/min under air flow (100 mL/min).

Scanning electron microscopy (SEM)

Scanning electron microscopy (SEM) images for the prepared catalysts were collected on a Quanta 600 3D, incorporated with an X-ray energy Dispersive Spectrometer (EDS).

Transmission electron microscopy (TEM)

Transmission electron microscopy (TEM) images were collected on a JEOL JEM-1400 Flash equipment with accelerating voltage up to 200kV and OneView fast readout CMOS digital camera.

Nitrogen adsorption-desorption isotherms

Nitrogen adsorption-desorption isotherm measurements were collected at 77K and 298K, respectively, using an IGA001 and ASAP2020 surface area analyzer. Before the measurement, the sample was degassed at 100 °C for 12 h before data acquisition.

Fourier-Transform InfraRed (FTIR) spectroscopy

FT-IR spectra were recorded on a Perkin-Elmer 283 using KBr as a matrix for the pellet samples.

Nuclear Magnetic Resonance (NMR) spectroscopy

Nuclear magnetic resonance (NMR) spectra were acquired on a Bruker AV-300 spectrometer (Bruker Corporation, Billerica, MA, USA), running at 300 MHz for ^{1}H. Chemical shifts (δ) are reported in ppm relative to residual solvent signals ($CDCl_3$: 7.26 ppm for ^{1}H-NMR). ^{13}C solid-state nuclear magnetic resonance was acquired on a Bruker AV-400 spectrometer coupled to a multinuclear probe ($^{15}N^{31}P$) CPMAS with triple channel (BL4 X/Y/1H) for a 4 mm rotor at room temperature, using 1k scans and 12 kHz of turning speed. The ^{1}H excitation pulse used is $\pi/2 \times 2.75$ μs, and the contact pulse is 3 ms.

X-ray photoelectron spectroscopy (XPS) studies

X-ray photoelectron spectroscopy (XPS) measurements were carried out under ultra-high vacuum conditions (10^{-10} mbar) using a SPECS GmbH electron spectroscopy system provided with a PHOIBOS 150 9MCD analyzer and a double anode X-ray source (Al/Mg). The samples were prepared as pellets.

Diffuse reflectance (DR) UV-VIS spectroscopy

Diffuse reflectance (DR) UV-VIS spectra were obtained on a PerkinElmer UV-Vis Spectrometer. The spectra were recorded at room temperature in the range of 200–800 nm.

X-ray Diffraction (XRD)

Crystallinity was studied through X-ray diffraction measurements, conducted with a Panalytical Empyrean and Bruker D8 diffractometers at 45 kV and 40 mA, using Cu-Kα radiation (λ = 1.5418 Å).

2. Computational details

All calculations were carried out using the Orca 6.1.0 software package[1] within the framework of density functional theory and its time dependent version (TD-DFT). The CAM-B3LYP functional was employed in combination with the def2-SVP basis set[2] and the corresponding Coulomb-fitted auxiliary basis set[3], including D3BJ dispersion corrections.[4,5] All the TD-DFT calculations were performed within the Tamm-Dancoff approximation.[6]

Electrocatalysis

Ground state geometry optimizations were performed for the tetramer structures of PAF-Phen and PAF-Phen-BT. Based on these structures, we computed the energies of the radical cations to estimate the vertical ionization potentials (VIE). The radical cations were also optimized, and the vertical energies of the neutral species were calculated to obtain the electron attachment energies (EAE). Due to their size, these systems likely exhibit multiple minima on their potential energy surfaces. In this work, only the most stable ground state minima were considered under the following assumptions: (1) the optimized structures are essentially planar, and representative of the solid; thus out-of-plane distorted conformations were neglected; (2) structure flexibility is mainly associated with the linker rotation, which is expected to be hindered in the solid state, reducing the number of accessible conformations; (3) the electronic and optical properties are not expected to be significantly affected by small structural changes, allowing a single minimum to be considered representative. Although this simplified model does not account for long-range periodicity or interlayer stacking effects, it is expected to capture the essential features of the materials, as supported by the good agreement between computed and experimental energy gap values and absorption spectra.

To explain the differences in the catalytic behavior, we computed the adsorption energies of a single oxygen molecule on the four binding configurations shown in Figure 9. The adsorption energies (E_{ads}) were calculated using the counterpoise method as in Equation 1, where E_{PAF-O2} is the energy of PAF-Phen or PAF-Phen-BT - O_2 complexes, E_{PAF} is the energy of the PAF fragments on the optimized PAF-O_2 complexes including the basis functions of O_2, and E_{O2} is the energy of O_2 in the PAF-O_2 complexes including the basis functions of the PAFs.

$$E_{ads} = E_{PAF-O_2} - E_{PAF} - E_{O_2} \quad \text{Eq.1}$$

Photocatalysis

The absorption spectra of the lowest 20 excited states of the PAF-Phen and PAF-Phen-BT tetramers were calculated at the TD-CAM-B3LYP-D3BJ/def2-SVP level of theory.

Starting from the S_0 minima, we optimized the geometries of the lowest-lying excited states with the highest absorption intensities. These optimizations led to S_1 minima, were we computed the spin-orbit coupling matrix elements (H^{SO}) with the closest triplet states.[7] Minimum energy intersystem crossing (ISC) points between S_1 and the triplets with the largest ISC rate, estimated from the $(H^{SO}(S_1\text{-}T_n)/\Delta E(S_1\text{-}T_n))^2$ ratio, were optimized when the $\Delta E(S_1\text{-}T_n)$ exceeded 0.2 eV. Otherwise, the S_1 minimum itself was considered an ISC funnel. From these points, the triplet states we further optimized to reach the T_1 minima.

To rationalize the photosensitized degradation of para red by PAF-Phen, we also computed the energies of the T_1 of O_2, para red and furfuryl alcohol at the same level of theory.

3. Synthesis of the building block Phen-Br4 (1)

Scheme S1. *Synthesis of PAF´s building block Phen-Br$_4$*

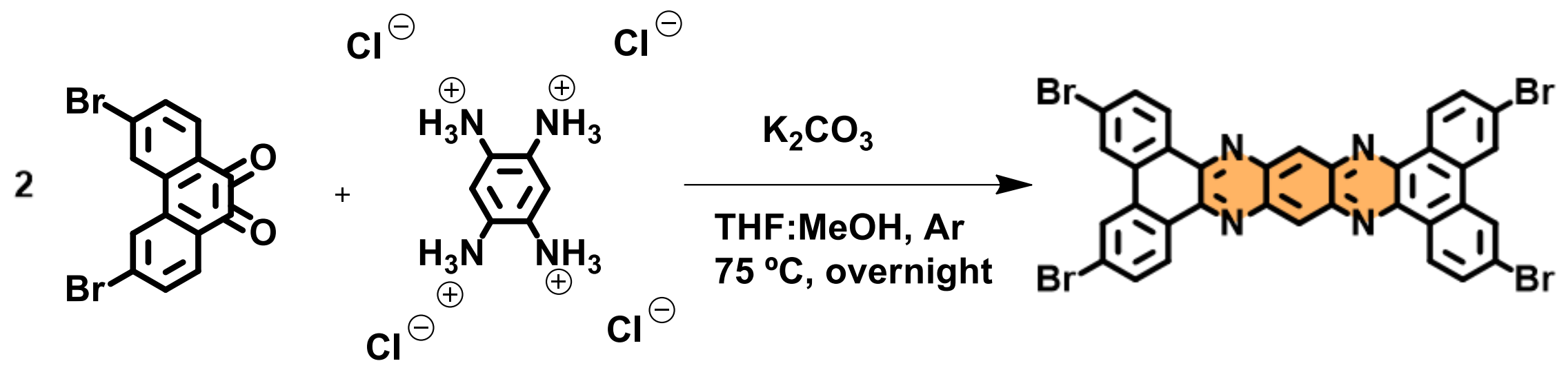

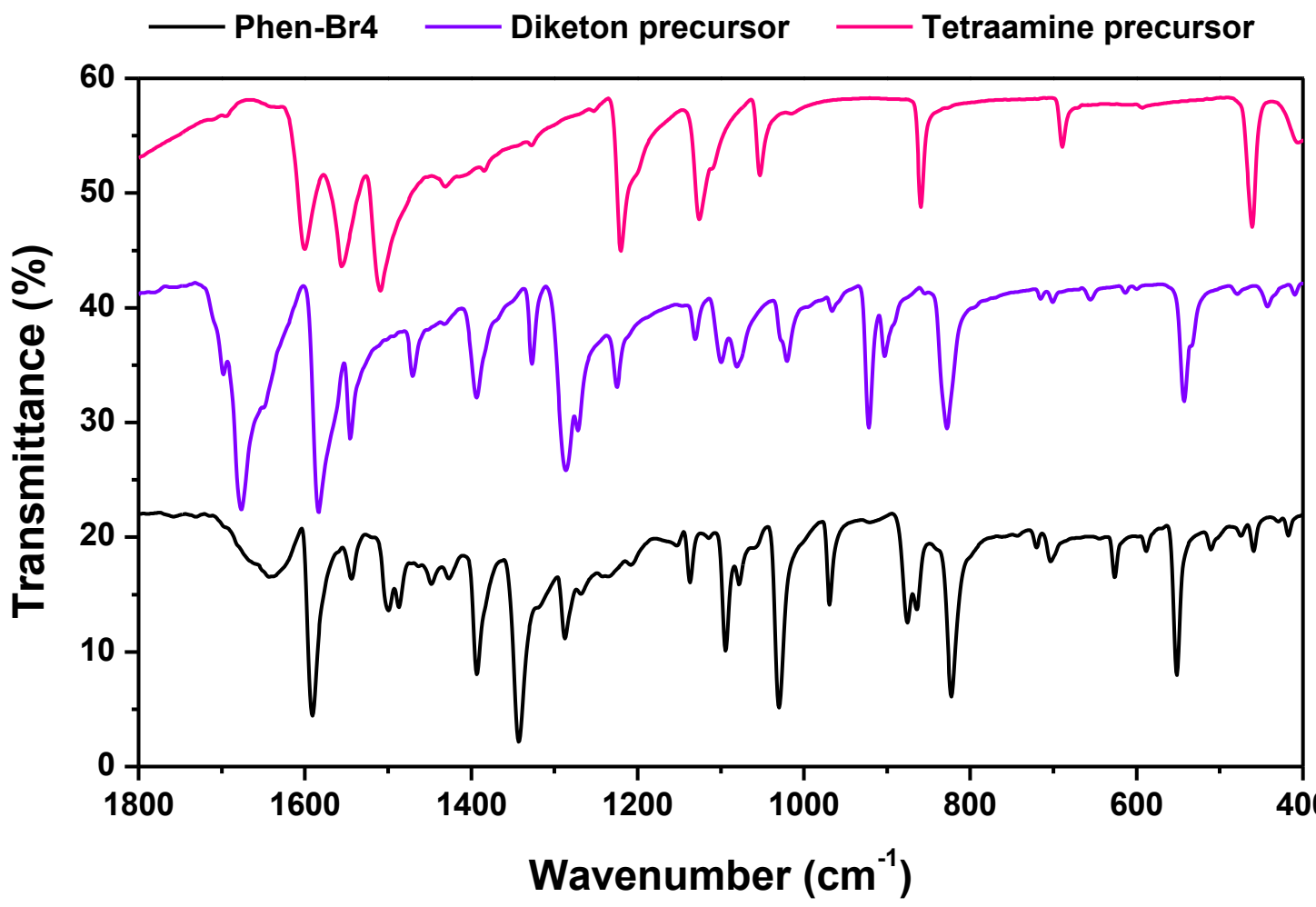

Figure S1. *FTIR spectra (KBr) of A) PAF's building block (PAF-Phen-Br$_4$) and its amine and ketone precursors.*

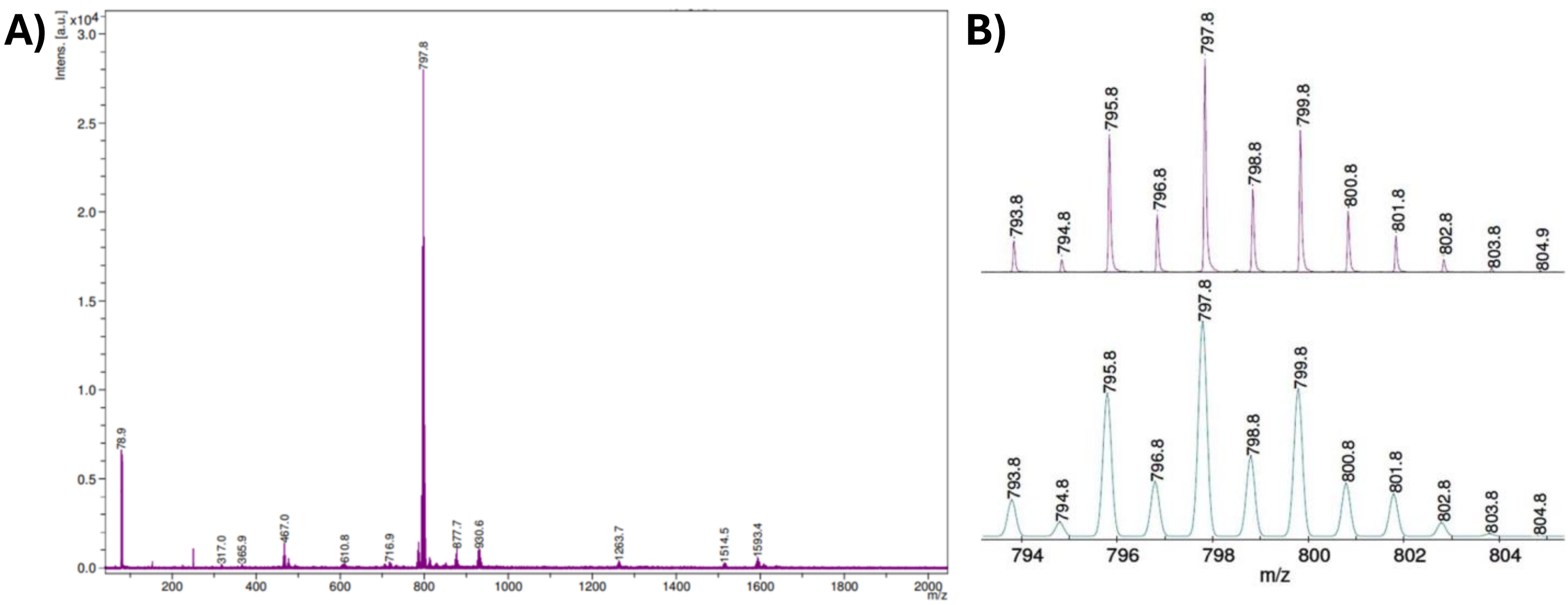

Figure S2. *A) MALDI-TOF Mass spectrum (DCTB matrix, negative ion detection) of building block* **1** and *B) its theoretical (bottom) and experimental (top) isotopic distributions.*

4. Synthesis of PAF materials

4.1 Synthesis of PAF-Phen

Scheme S2. *Synthesis of* ***PAF-Phen*** *material.*

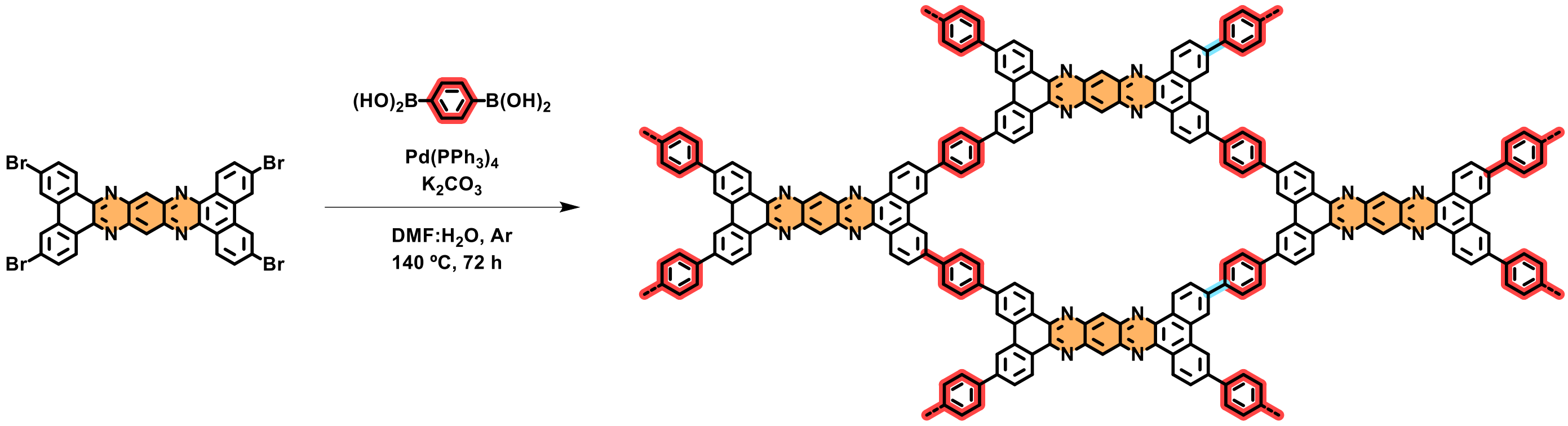

4.2 Synthesis of PAF-Phen-BT

Scheme S3. *Synthesis of* ***PAF-Phen-BT*** *material.*

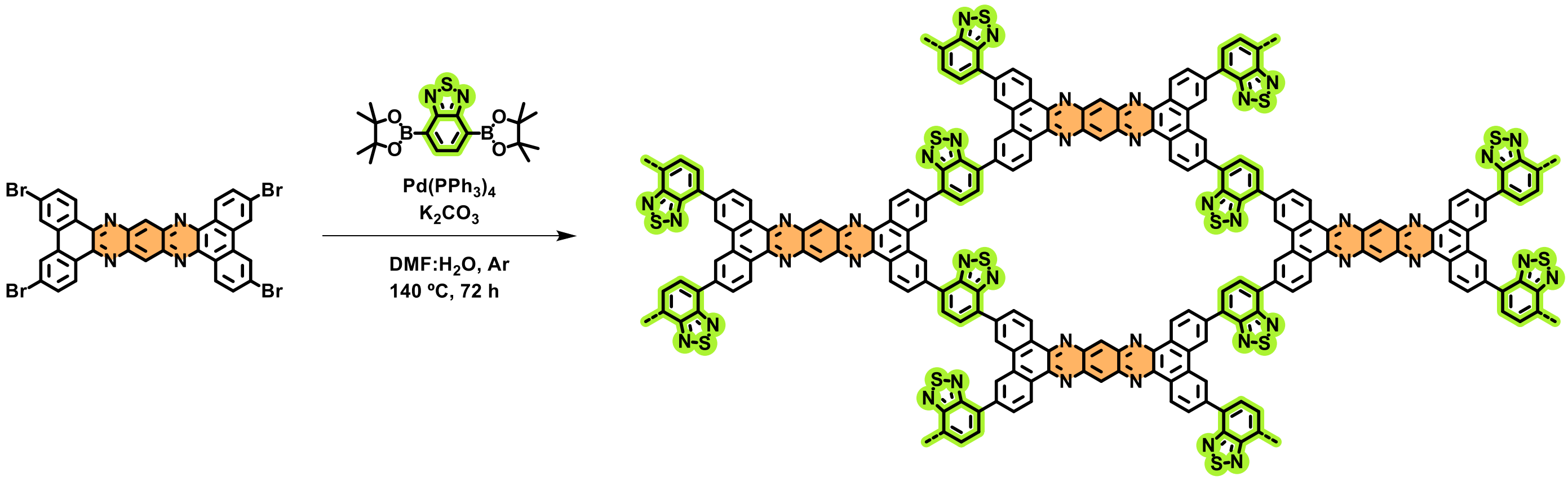

Table S1. *Synthetic conditions of PAFs materials.*

Material	Building Block	Boronic precursor	DMF:H2O volume	Mass obtained
**PAF-Phen**	150 mg (0.17 mmol)	58 mg (0.35 mmol)	25.2 mL	114 mg (91 % yield)
**PAF-Phen-BT**	150 mg (0.17 mmol)	146 mg (0.35 mmol)	25.2 mL	111 mg (75 % yield)

Table S2. *Elemental analysis of PAF materials*.

Material	$\%C_{exp}$	$\% C_{teo}$	$\%H_{exp}$	$\% H_{teo}$	$\%N_{exp}$	$\% N_{teo}$	$\%S_{exp}$	$\%S_{teo}$
**Phen-Br$_4$·4HCl**	41.90	43.26	1.89	1.92	5.90	5.94		
**PAF-Phen**	71.31	87.6	3.66	3.52	7.02	8.88		
**PAF-Phen-BT**	67.7	73.98	3.47	2.43	12.87	15.01	8.09	8.59

The experimentally determined elemental compositions are in reasonable agreement with the expected values. The slightly lower values observed for the polymeric materials are attributed to the well-known difficulties associated with the complete combustion of highly crosslinked and thermally robust porous organic polymers during elemental analysis. In the case of Phen-Br$_4$, the elemental analysis is consistent with the presence of HCl, likely originating from the tetrahydrochloride tetraamine precursor and retained through protonation and/or strong hydrogen-bonding interactions.

5. Comparative FTIR spectra of PAF materials and their respective precursors.

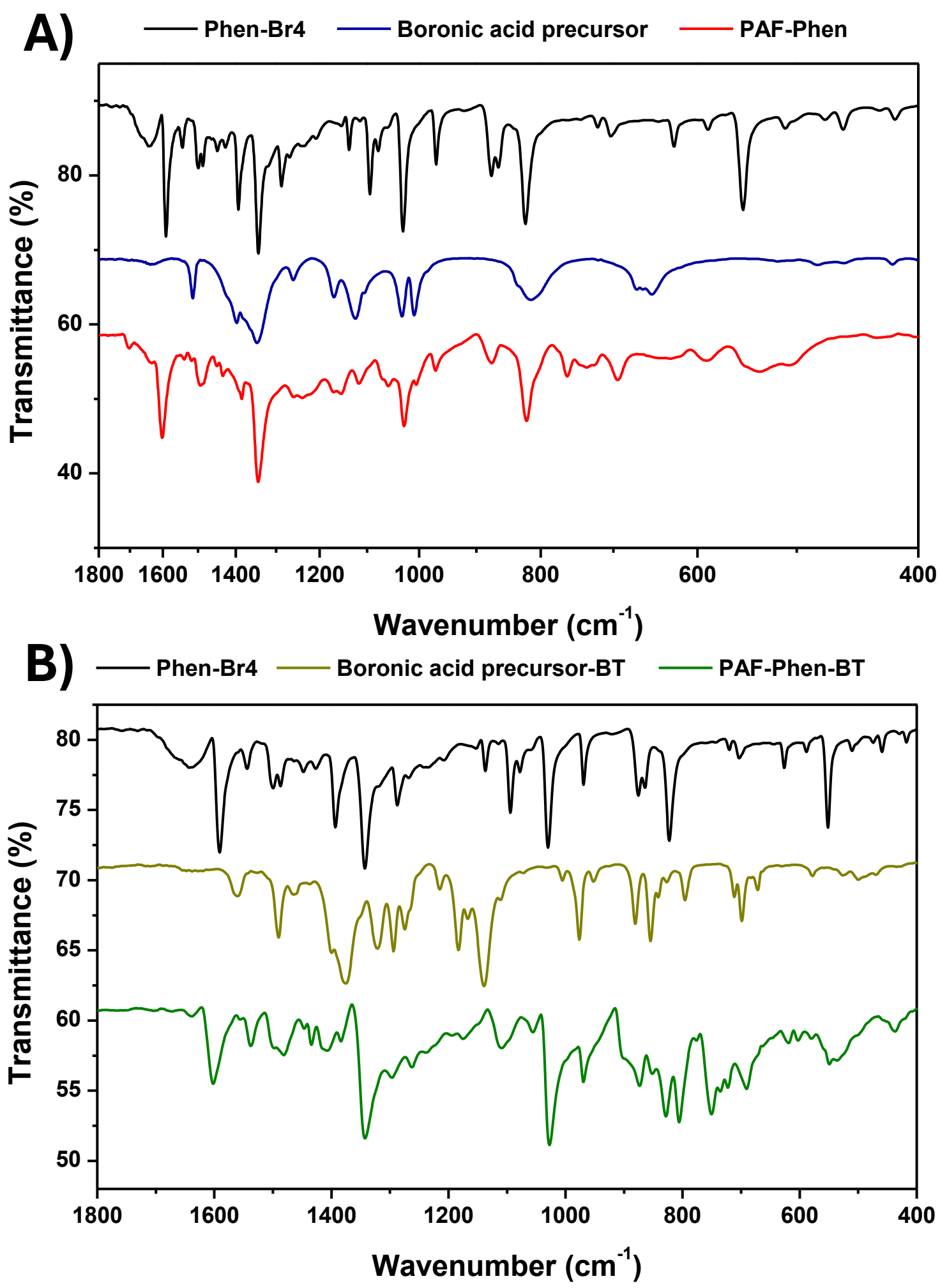

Figure S3. *FTIR spectra (KBr) of A) PAF-Phen and its precursors and B) PAF-Phen-BT and its precursors.*

6. XPS analysis of the materials

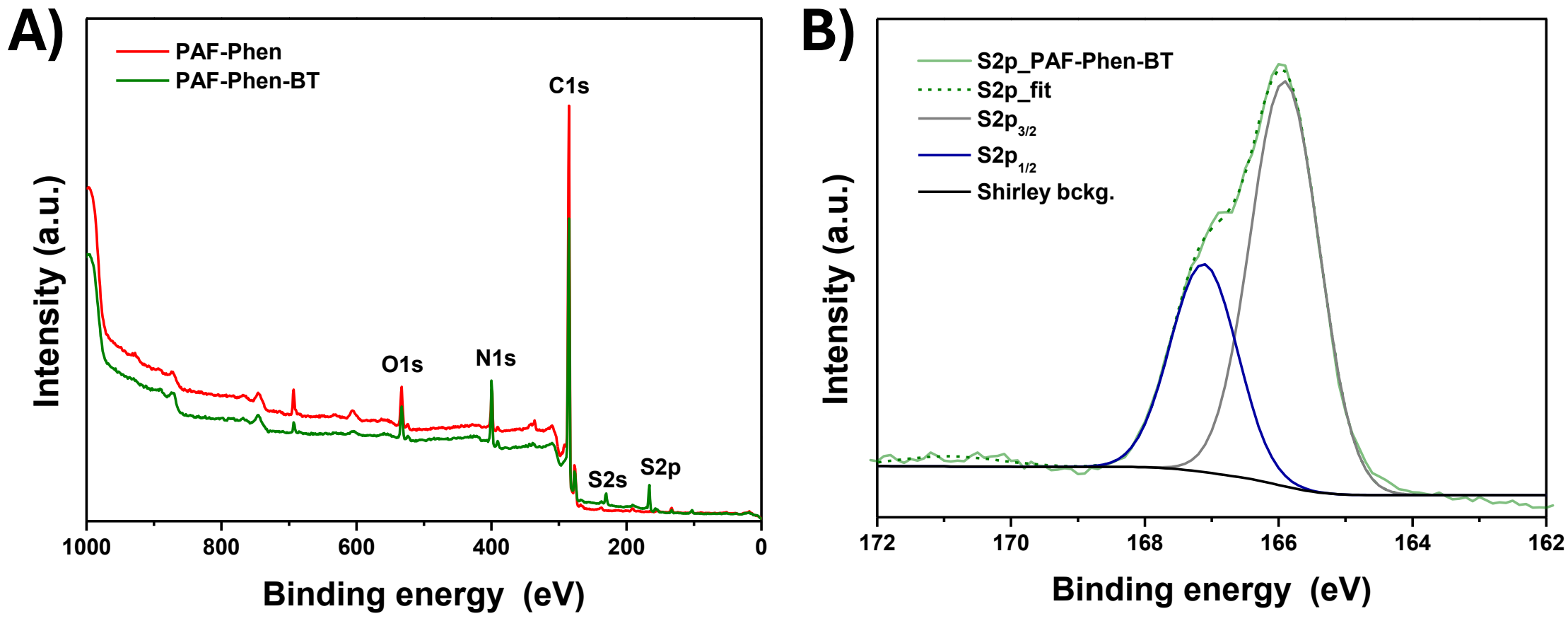

Figure S4. *A) Survey XPS spectra, B) S2p XPS spectra of PAF-Phen-BT and its fit peaks.*

7. Kubelka Munk calculations for the optical band gap of the materials

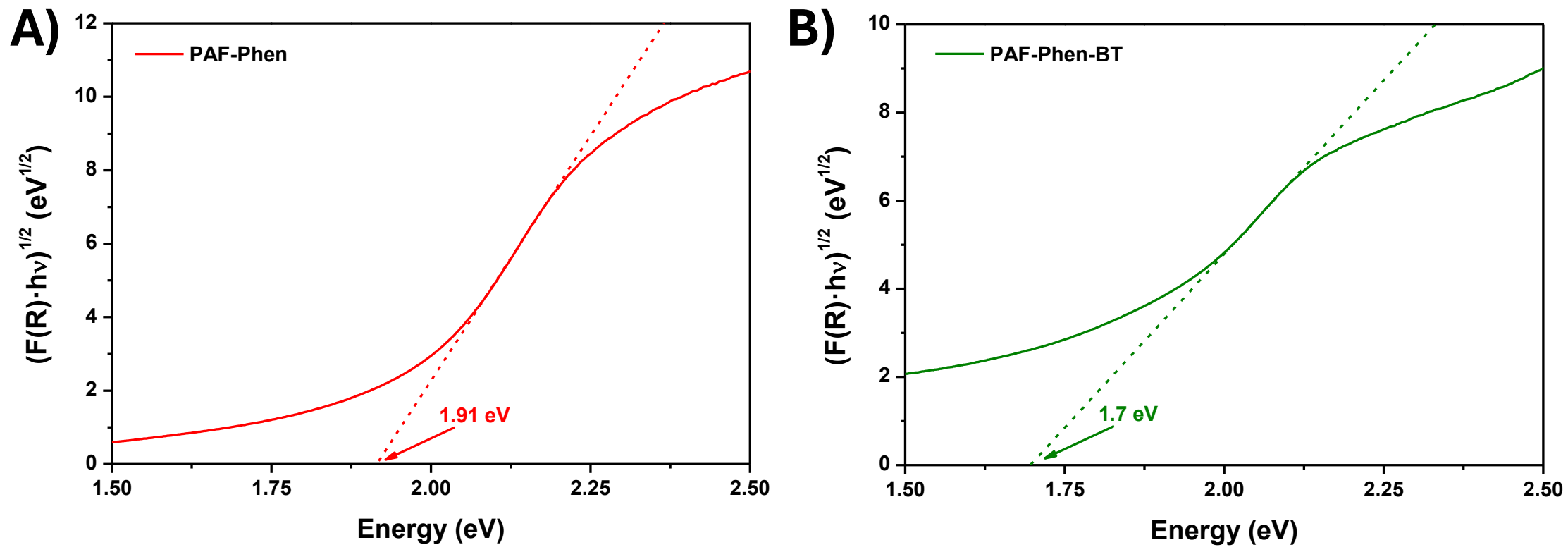

Figure S5. *Linear fit for the K-M indirect band gap calculation for A) PAF-Phen and B) PAF-Phen-BT materials.*

8. Analysis of the XPS valence band region

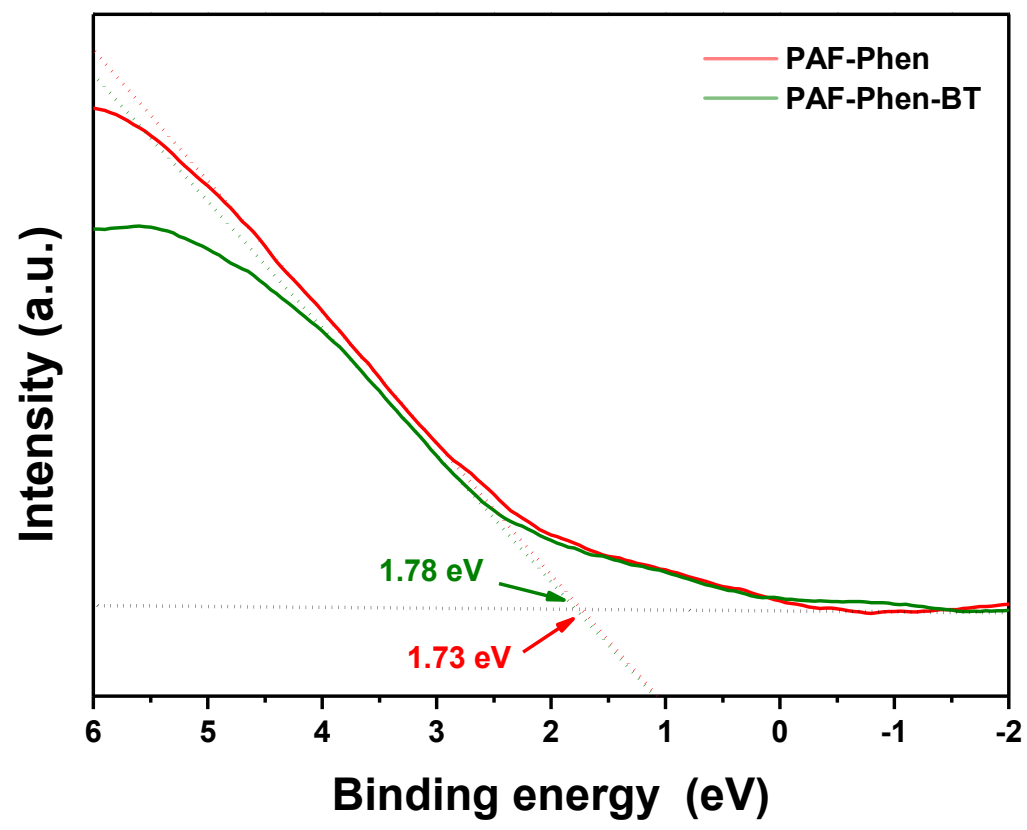

Figure S6. *Fit of the valence band region of the XPS spectra of PAF materials*

9. X-ray Diffraction of the materials

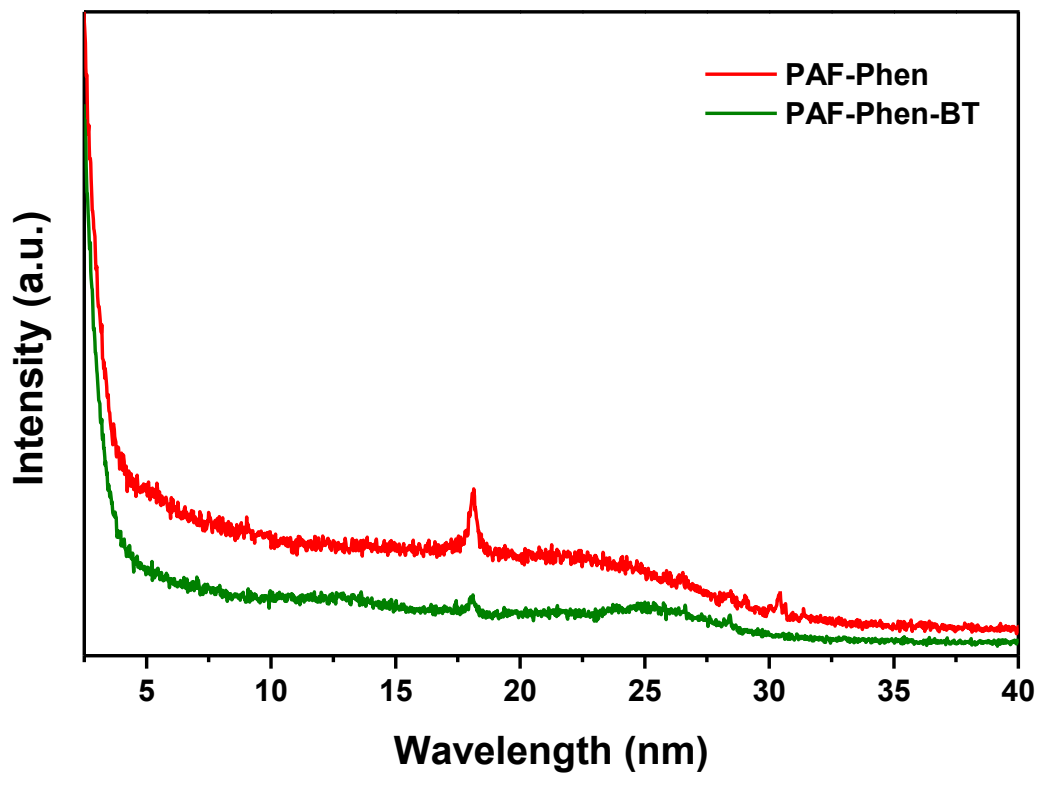

Figure S7. *X-ray diffractograms of PAF materials.*

10. Nitrogen Isotherm of the materials

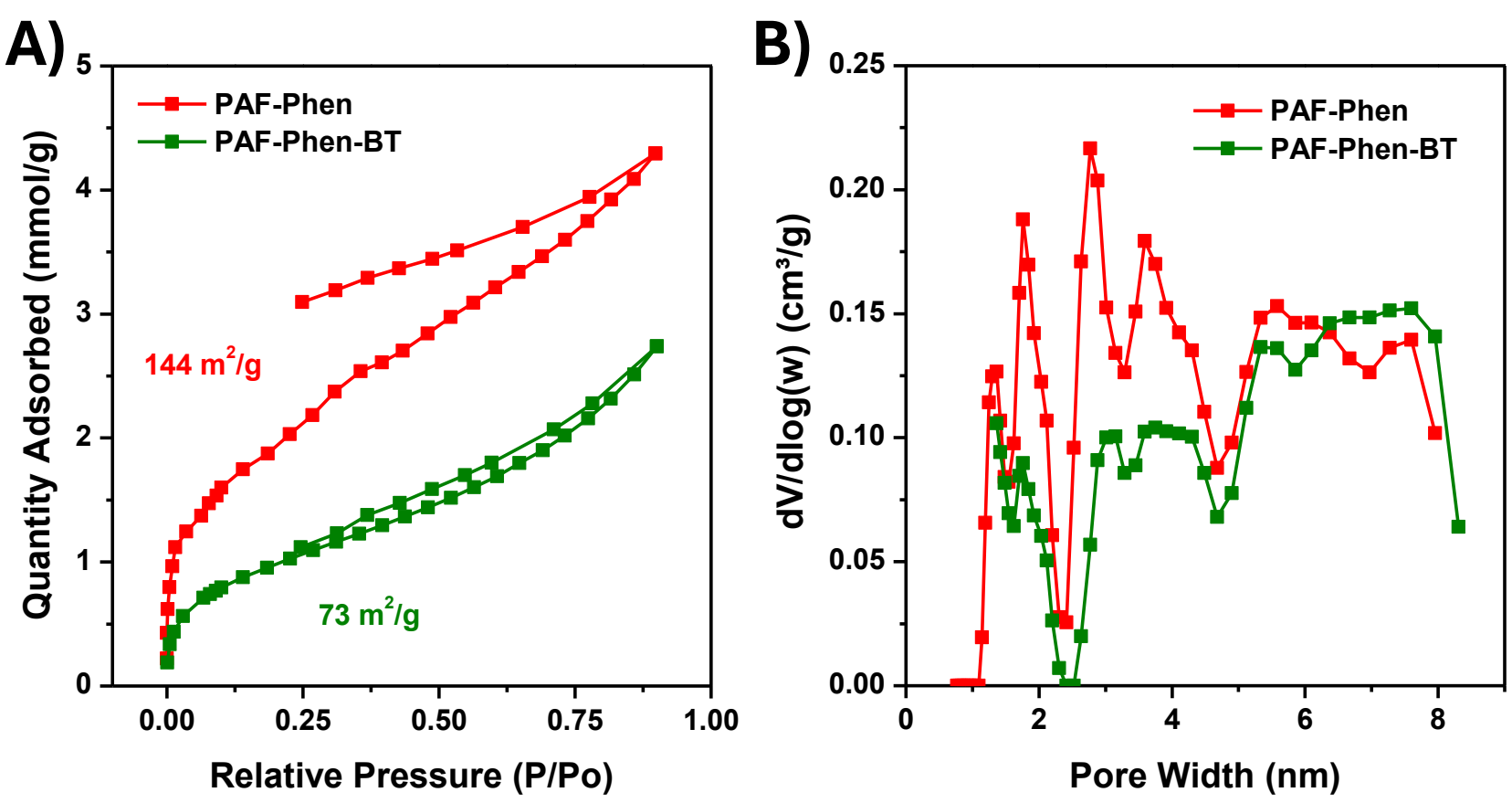

Figure S8. *A) Nitrogen adsorption-desorption isotherms and B) NLDFT pore size distributions for PAF-Phen and PAF-Phen-BT materials.*

11. Thermogravimetric analysis of the materials

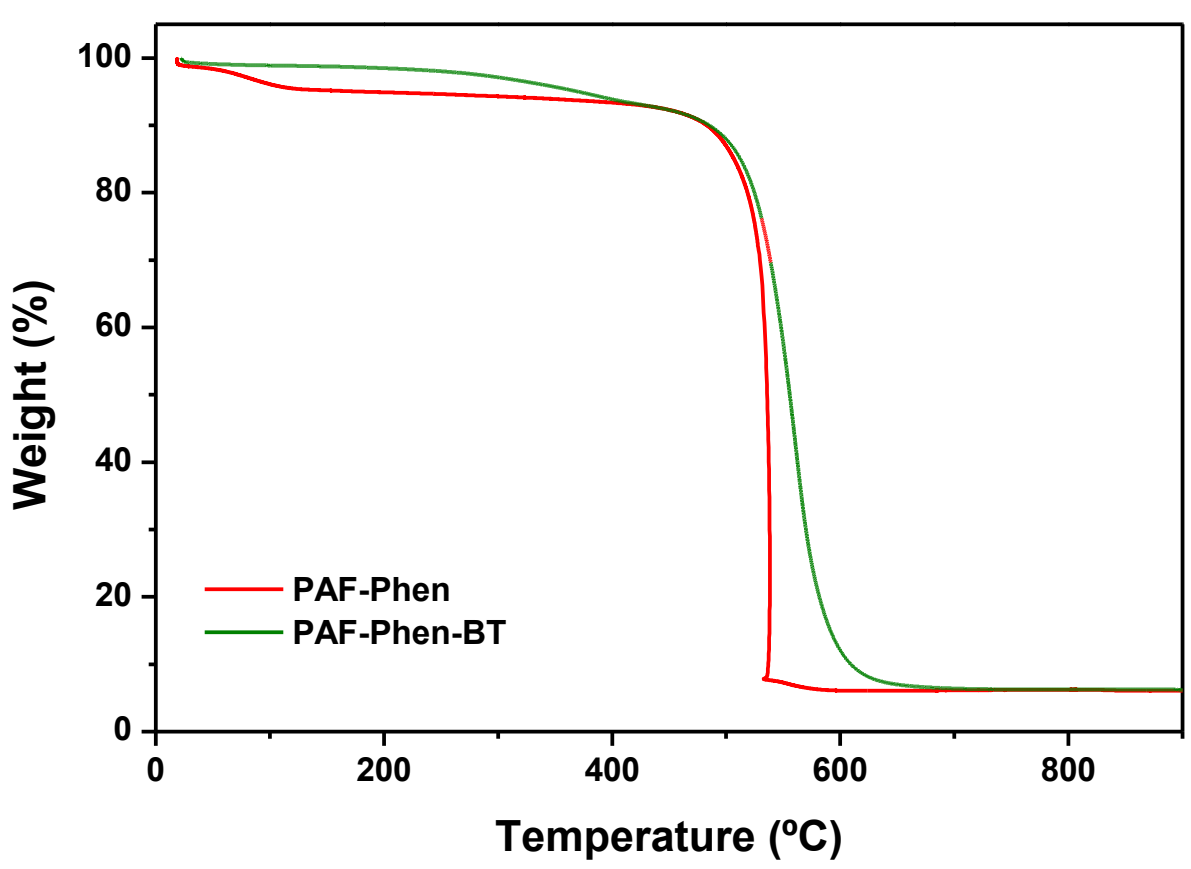

Figure S9. *Thermogravimetric analysis of PAF materials.*

12. Photodegradation of para red (PR)

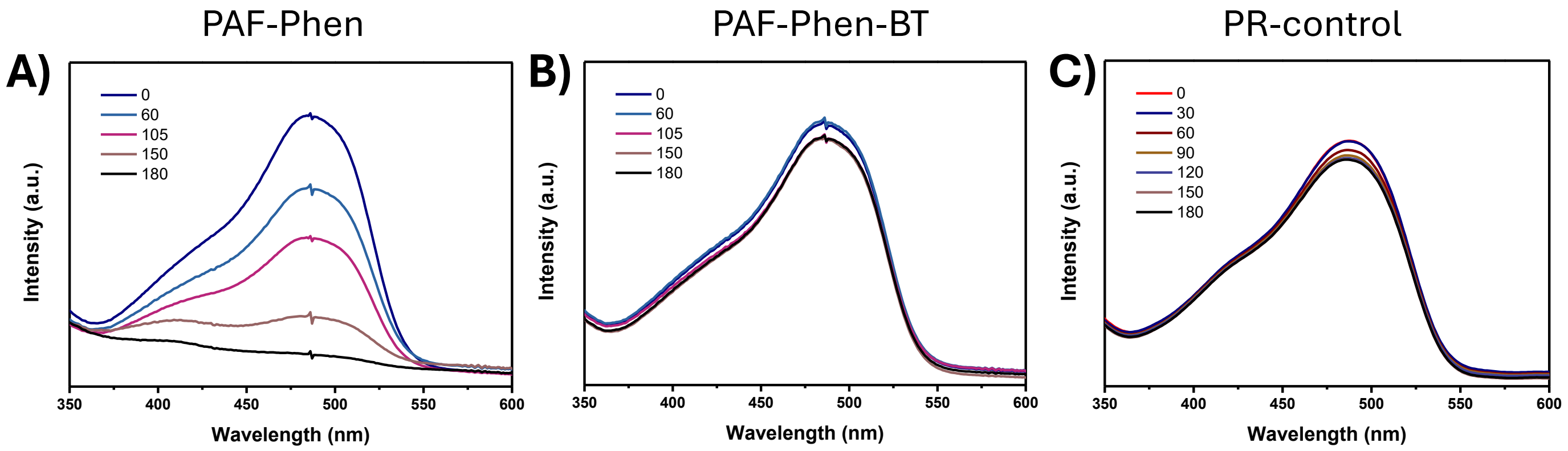

Figure S10. *UV-Vis spectra of the kinetic study of the photodegradation of PR using A) PAF-Phen, B) PAF-Phen-BT and C) para red control without using catalyst.*

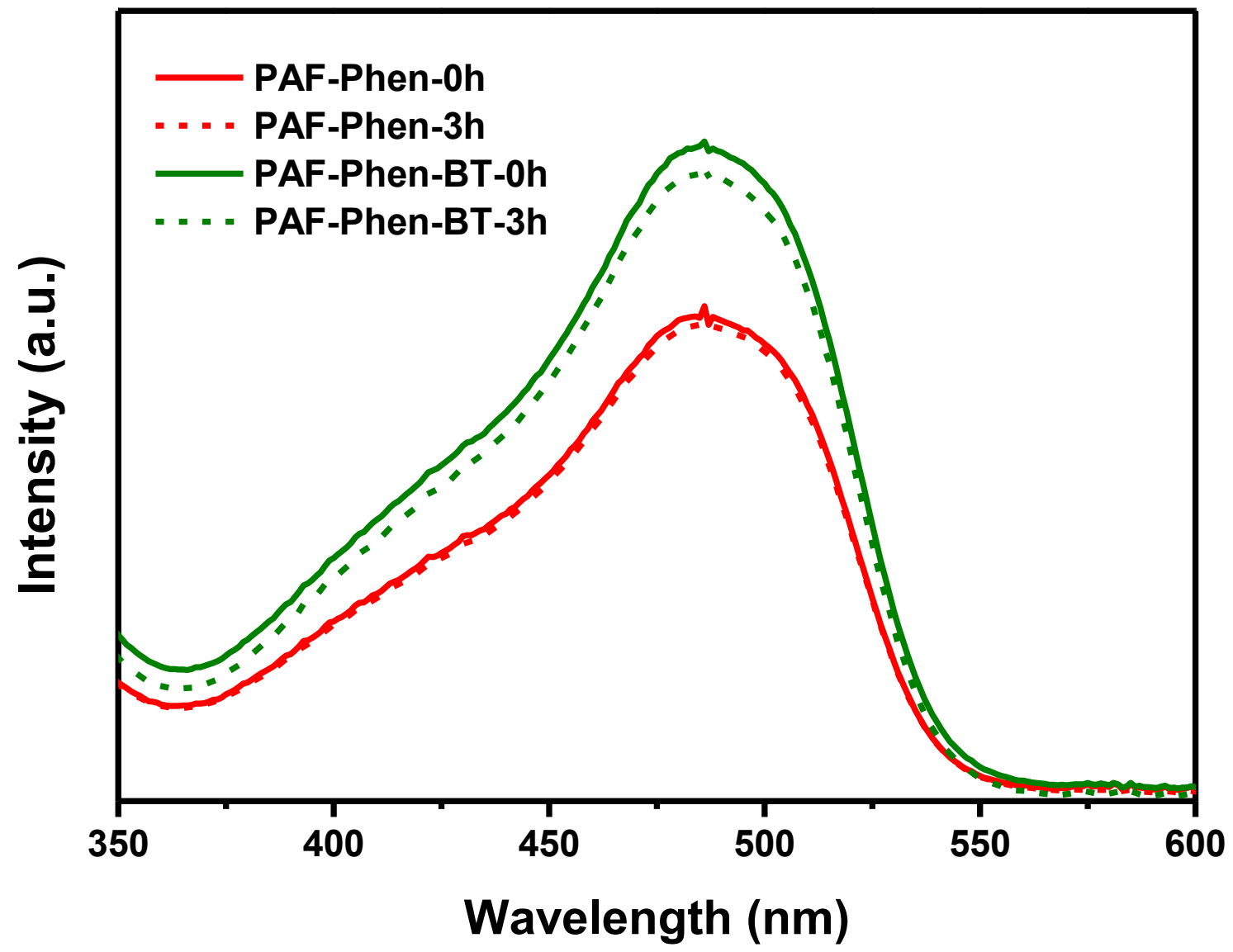

Figure S11. *UV-Vis spectra of PR before and after photodegradation in Ar atmosphere using PAF-Phen (red) and PAF-Phen-BT (green) catalysts.*

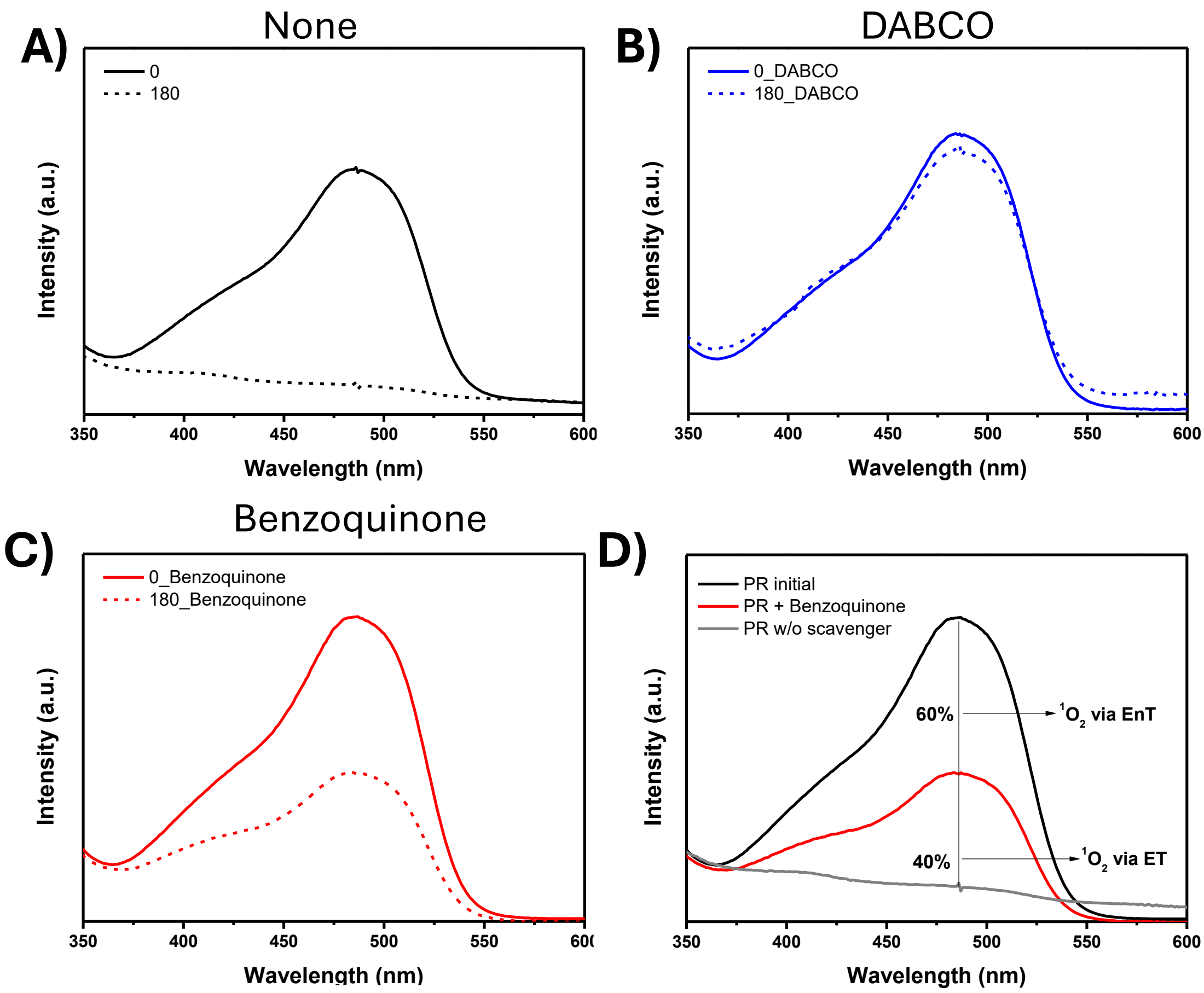

Figure S12. *UV-Vis spectra of PR before and after photodegradation using PAF-Phen material with A) any scavenger, B) DABCO and C) Benzoquinone scavengers and D) Comparative view of scavenger results where spectrum without scavenger has been normalized to the same initial concentration of PR.*

Table S3. *Photocatalytic organic materials for photodegradation of azo-dyes.*

Material name	Material type	Dye(s) Targeted	Light source	Photodegradation Efficiency (%)	Reference
**TpBD-COF (imine)**	Imine-linked material	Mordant Black 17 (150 ppm)	Sunlight	100% (60 min)	(1)
**TFA-TTA-COF (imine)**	Fluorinated COF	Eriochrome black T (10 ppm)	Blue LEDs (30 W)	9% (100 min)	(2)
		Eriochrome black A (10 ppm)		82 % (100 min)	
**TTO-COF (olefin)**	Extended conjugation of COFs	Methyl Orange (10 ppm)	Xenon lamp (300 W) (λ > 450 nm)	100% (20 min)	(3)
**COF_{A+C} (imine)**	N-rich COFs	Methyl Orange (10 ppm)	Xenon lamp (300 W) (λ > 450 nm)	100% (30 min)	(4)
**TFPT-PhenBM**	Vinylene-linked COFs	Para Red (10 ppm)	Blue LEDs (λ = 465 nm) (18 W/m^2)	100% (4.5 h)	(5)
**TFPB-PhenBM**				77% (4.5 h)	
**TFB-PhenBM**				65% (4.5 h)	
**AZO-TFP-BT-TFP COF**	donor–acceptor β-ketoenamine-linked COF	Methyl orange (12.5 ppm)	Visible light (λ > 450 nm)	100% (3 h)	(6)
**AZO-TFP-MBT-TFP COF**				97.02 ± 2.54 % (3 h)	
**CTFSx**	S-doped CTFs	Methyl orange (1 ppm)	Xenon lamp (400 W)	53.23% (6 h)	(7)
**PhCTFs**	Ph/Thiophene CTF	Congo red (20 ppm)	UV light	98% (30 min)	(8)
		Methyl orange (10 ppm)		93% (120 min)	
**PTCTFs**		Congo red (20 ppm)		91% (30 min)	
		Methyl orange (10 ppm)		83% (260 min)	
**CMP-P1**	Bifluorenylidene-based CMP	Methyl orange (60 ppm)	Xenon lamp (300 W) (λ > 450 nm)	60% (1 h)	(9)
**CMP-P2**				35% (1 h)	
**UPC-CMP-1**	Fe-porphyrin-based CMP	Congo red	Visible light	88.3% (120 s)	(10)
**PAF-Phen**	Bisphenazine-based PAF	Para Red (10 ppm)	Blue light LEDs (λ = 465 nm, 18 W/m^2)	100% (3 h)	This work

13. ^{1}HNMR spectra of FA oxidation experiments

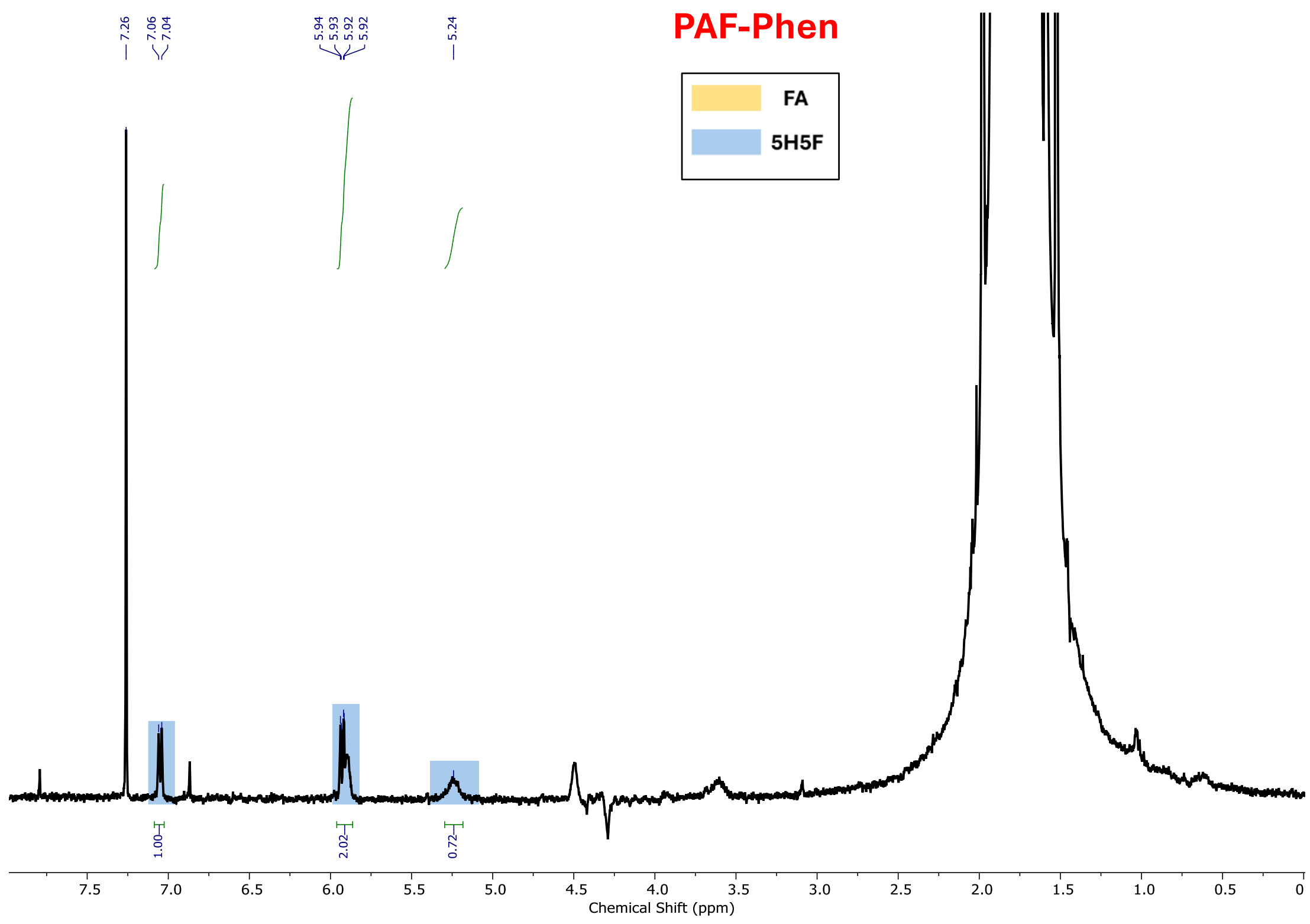

Figure S13. *^{1}H-NMR (300 MHz, $CDCl_3$) spectrum of crude sample from FA photocatalytic oxidation with PAF-Phen.*

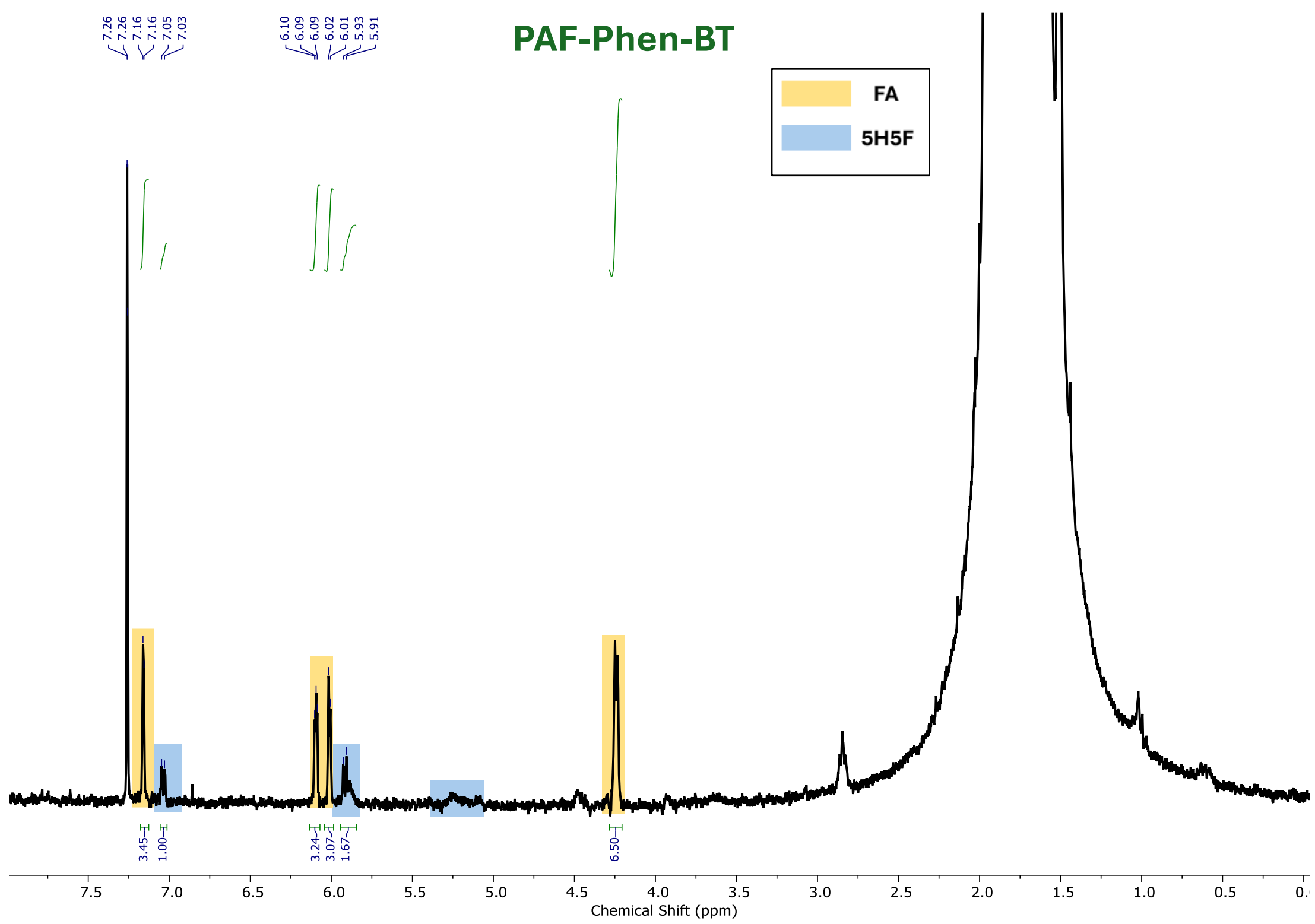

Figure S14. *^{1}H-NMR (300 MHz, $CDCl_3$) spectrum of crude sample from FA photocatalytic oxidation with PAF-Phen-BT.*

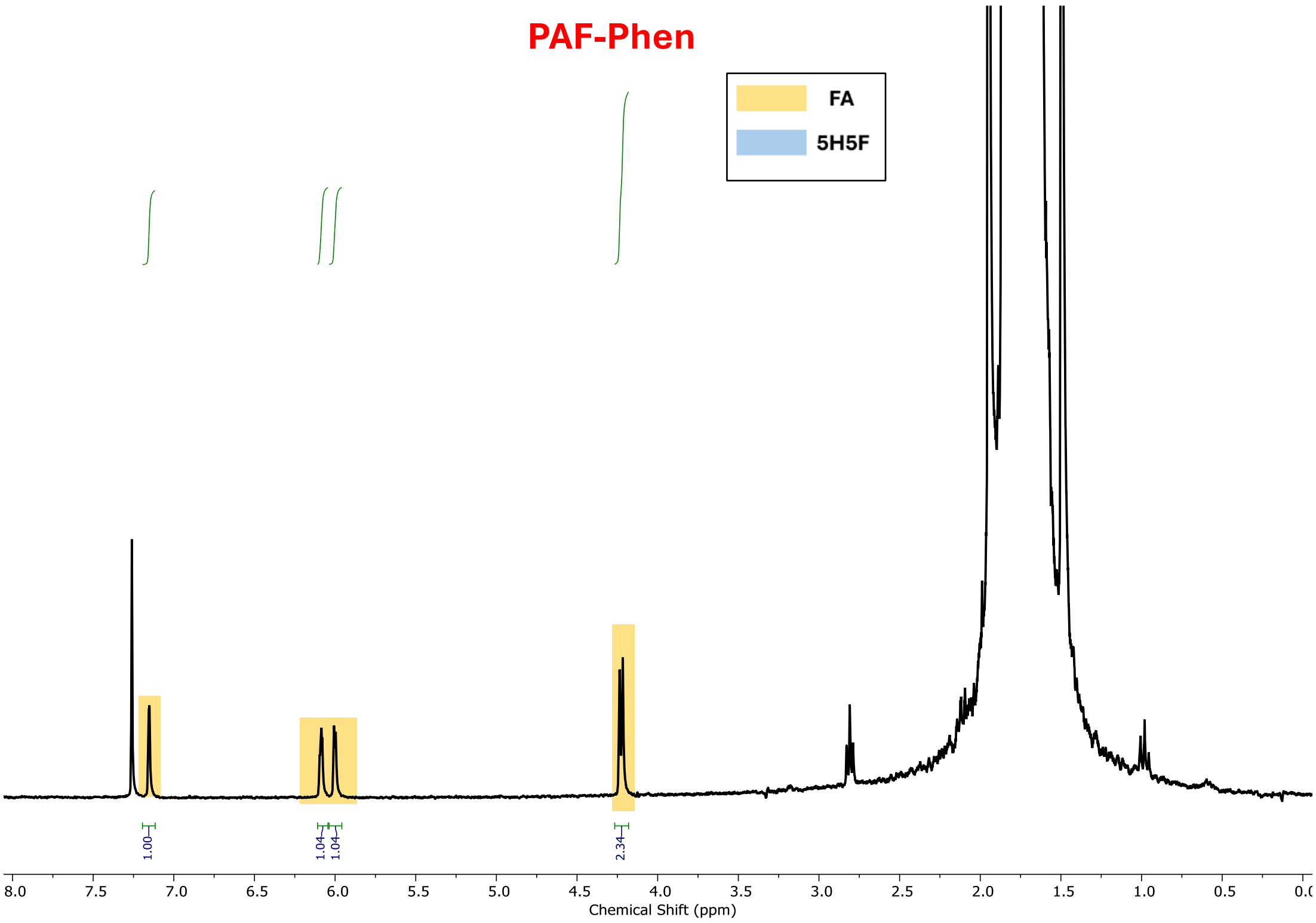

Figure S15. *^{1}H-NMR (300 MHz, $CDCl_3$) spectrum of crude sample from FA photocatalytic oxidation blank experiment in absence of catalyst.*

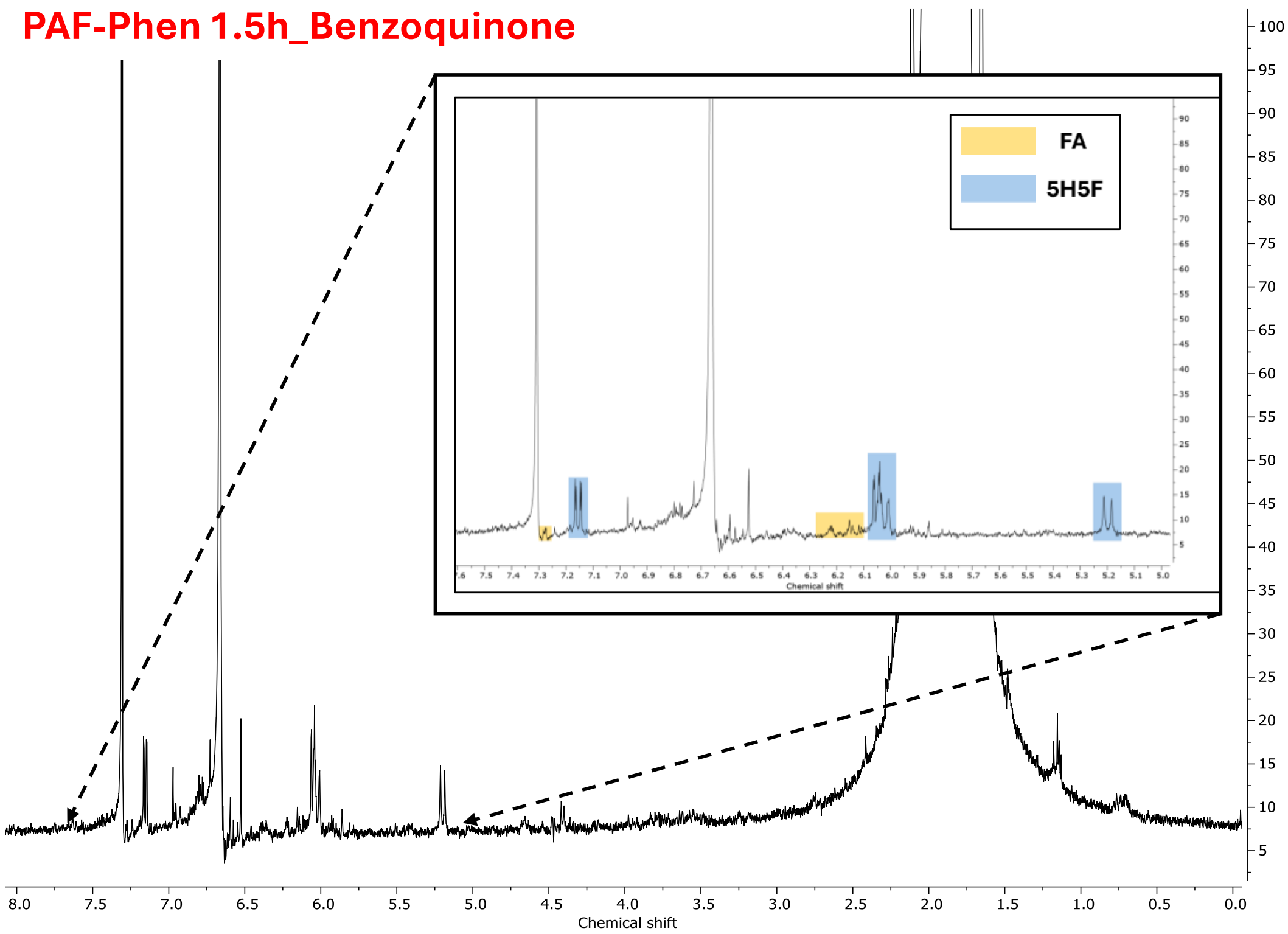

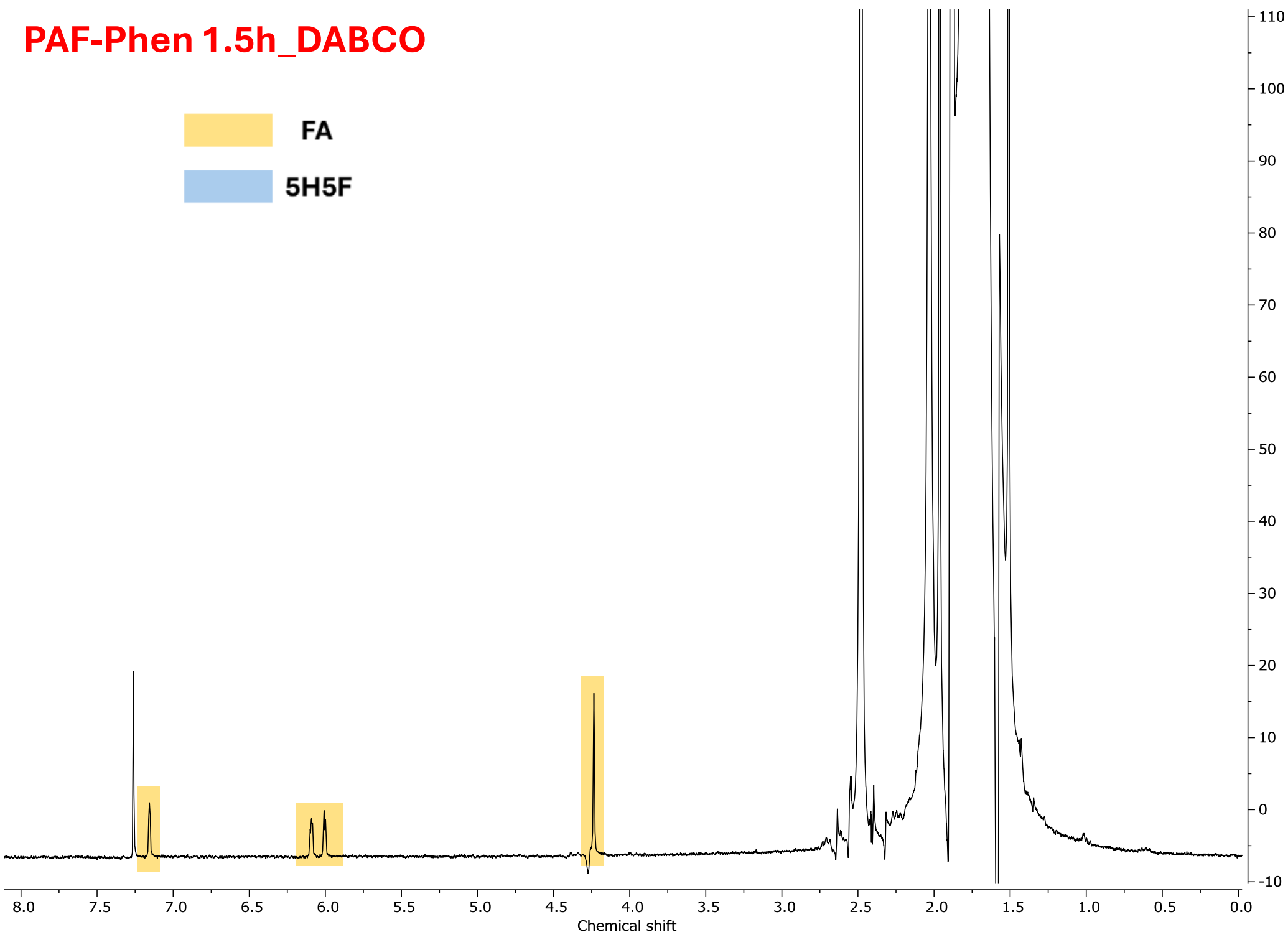

Figure S16. *^{1}H-NMR (300 MHz, $CDCl_3$) spectra of crude sample from FA photocatalytic oxidation in the presence of A) Benzoquinone and B) B) DABCO using PAF-Phen photocatalyst.*

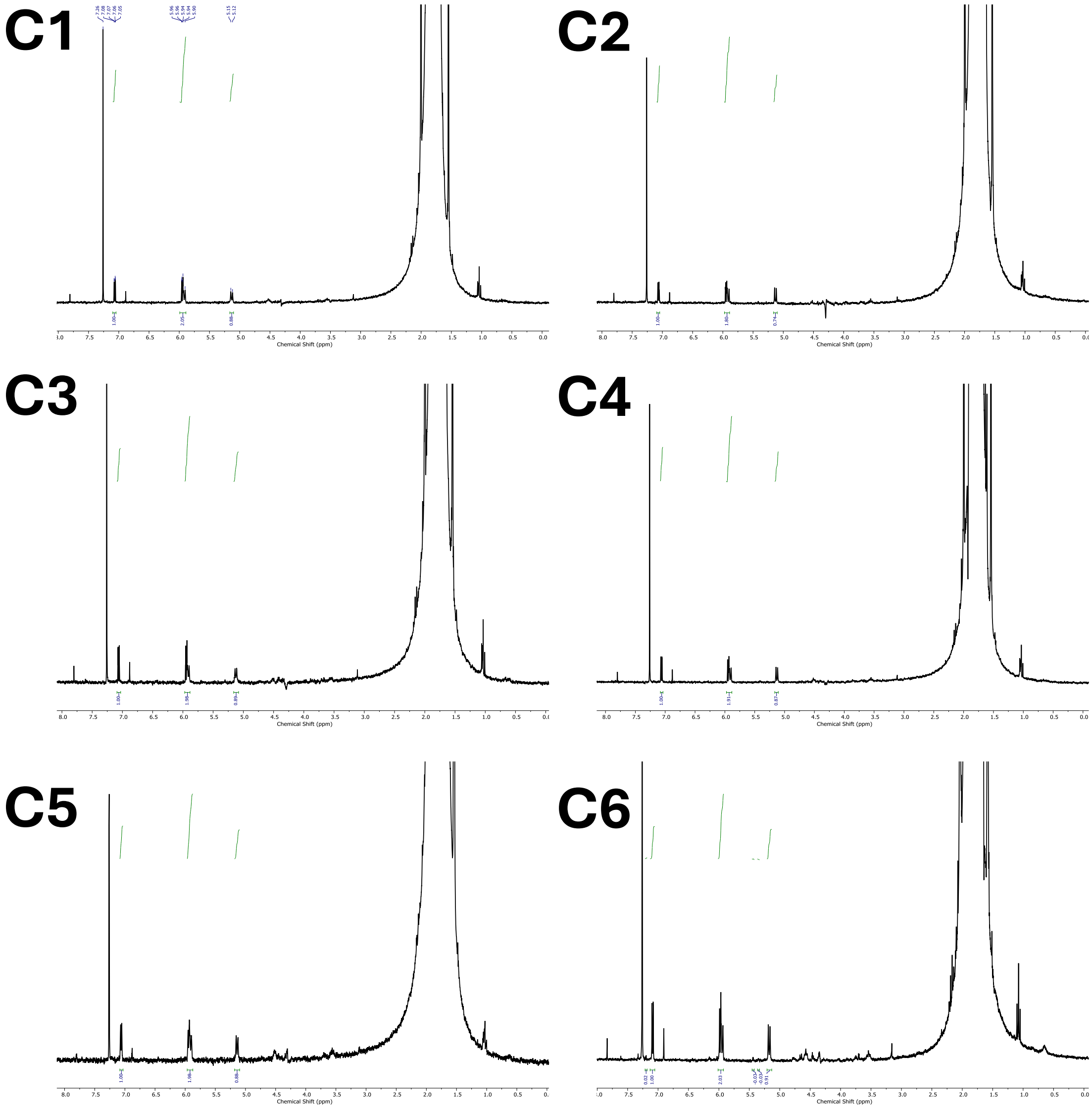

Figure S17. *^{1}H-NMR (300 MHz, $CDCl_3$) spectra of crude sample from each catalytic run (C1-6) of FA oxidation for the recyclability experiments with PAF-Phen as photocatalyst.*

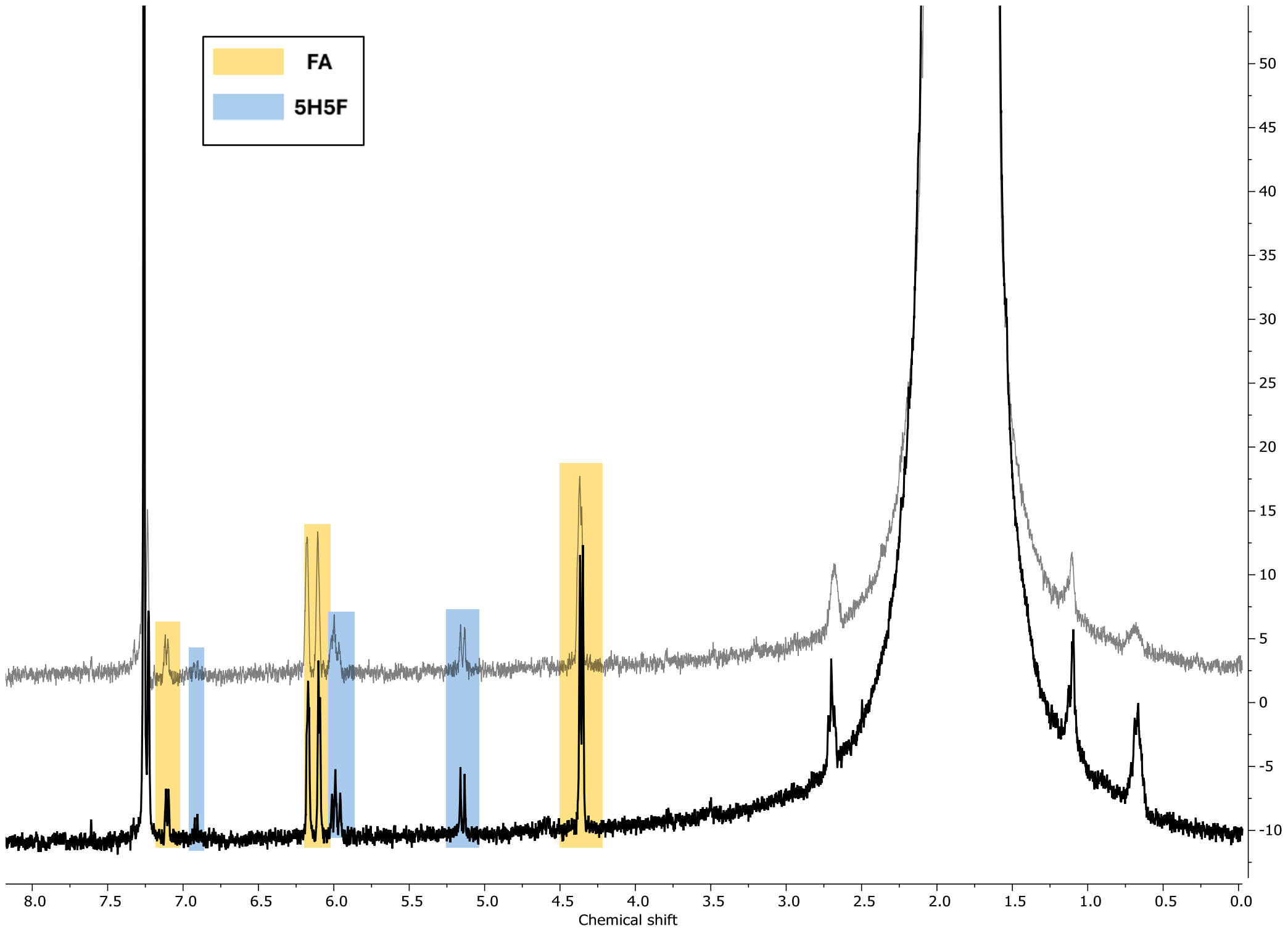

Figure S18. *^{1}H-NMR (300 MHz, $CDCl_3$) spectra of leaching experiment for FA photocatalytic oxidation using PAF-Phen after the first (black spectra) and the second (grey spectra) irradiation.*

14. FTIR and XPS spectra after recyclability test

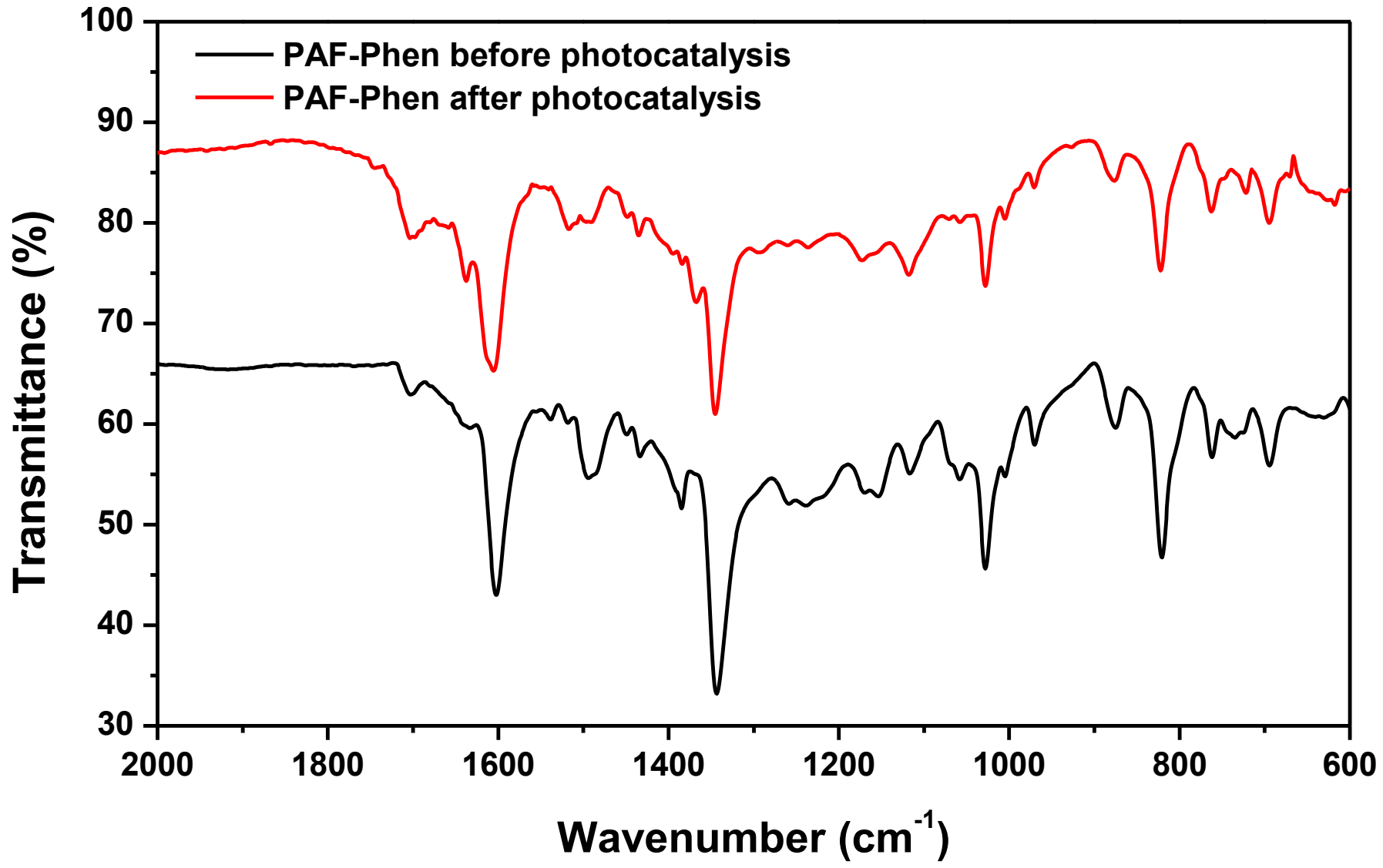

Figure S19. *A) FTIR spectra (KBr) before (black) and after (red) FA oxidation recyclability experiments using PAF-Phen material.*

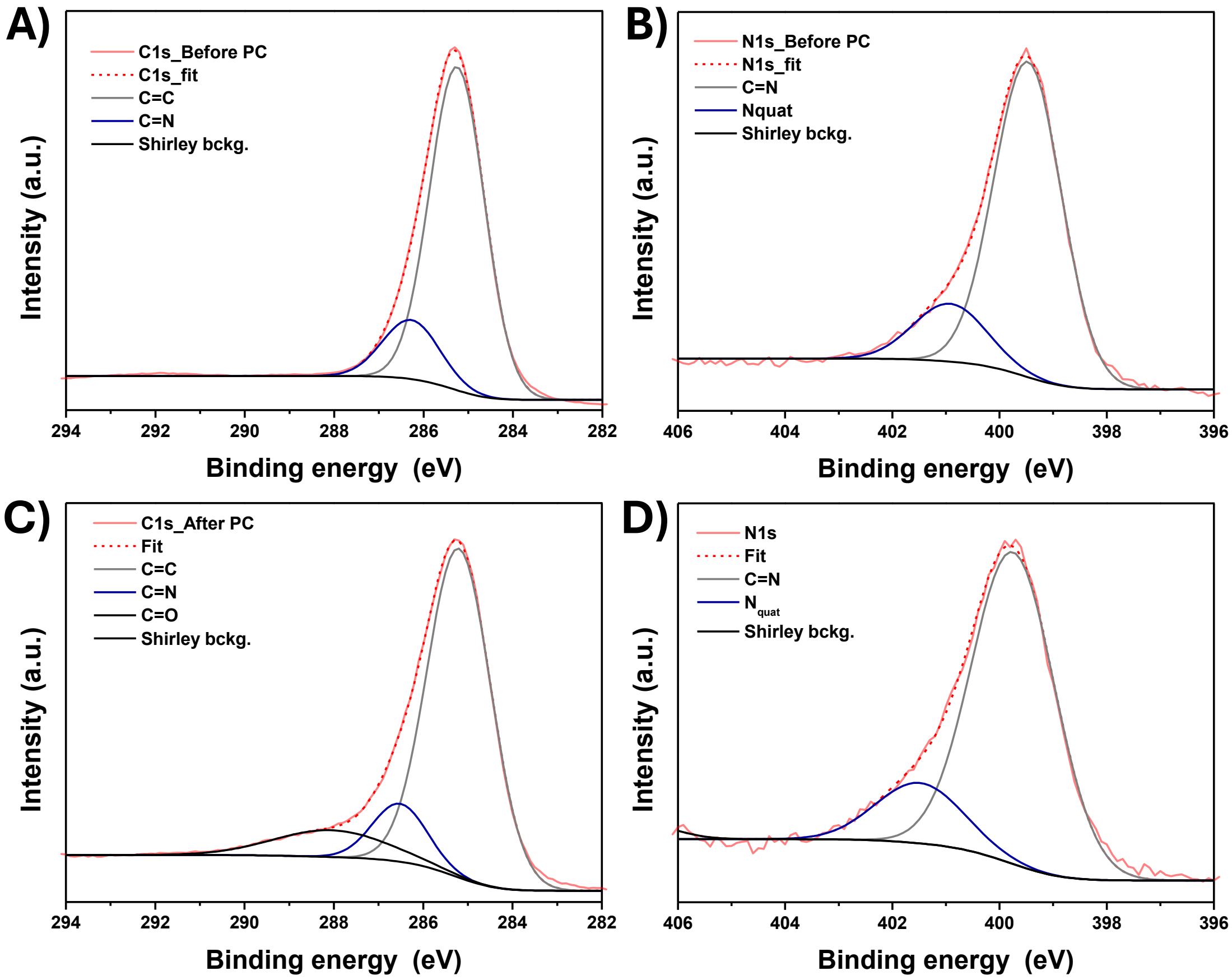

Figure S20. *XPS spectra of PAF-Phen material in the A) C1s and B) N1s regions before photocatalytic recyclability experiments and C) C1s and D) N1s regions after photocatalytic recyclability tests.*

15. Electrochemistry studies

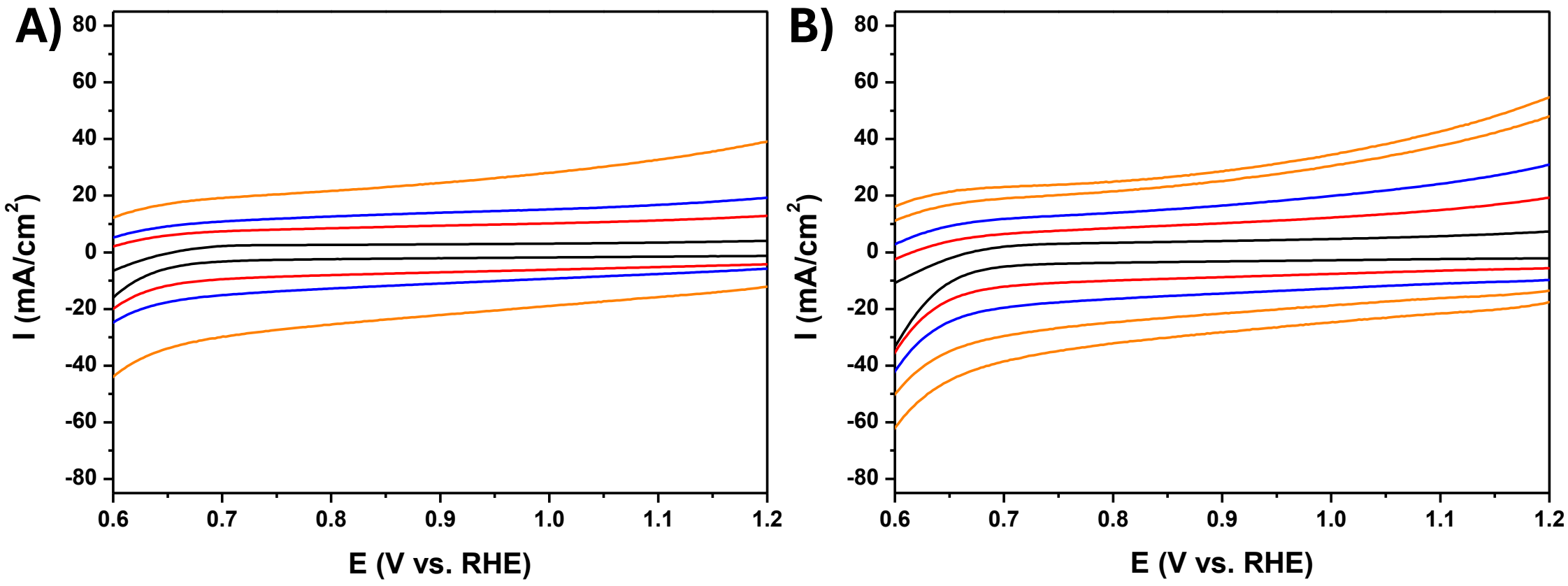

Figure S21. *Cyclic voltammetries under ORR conditions for A)PAF-Phen and B)PAF-Phen-BT.*

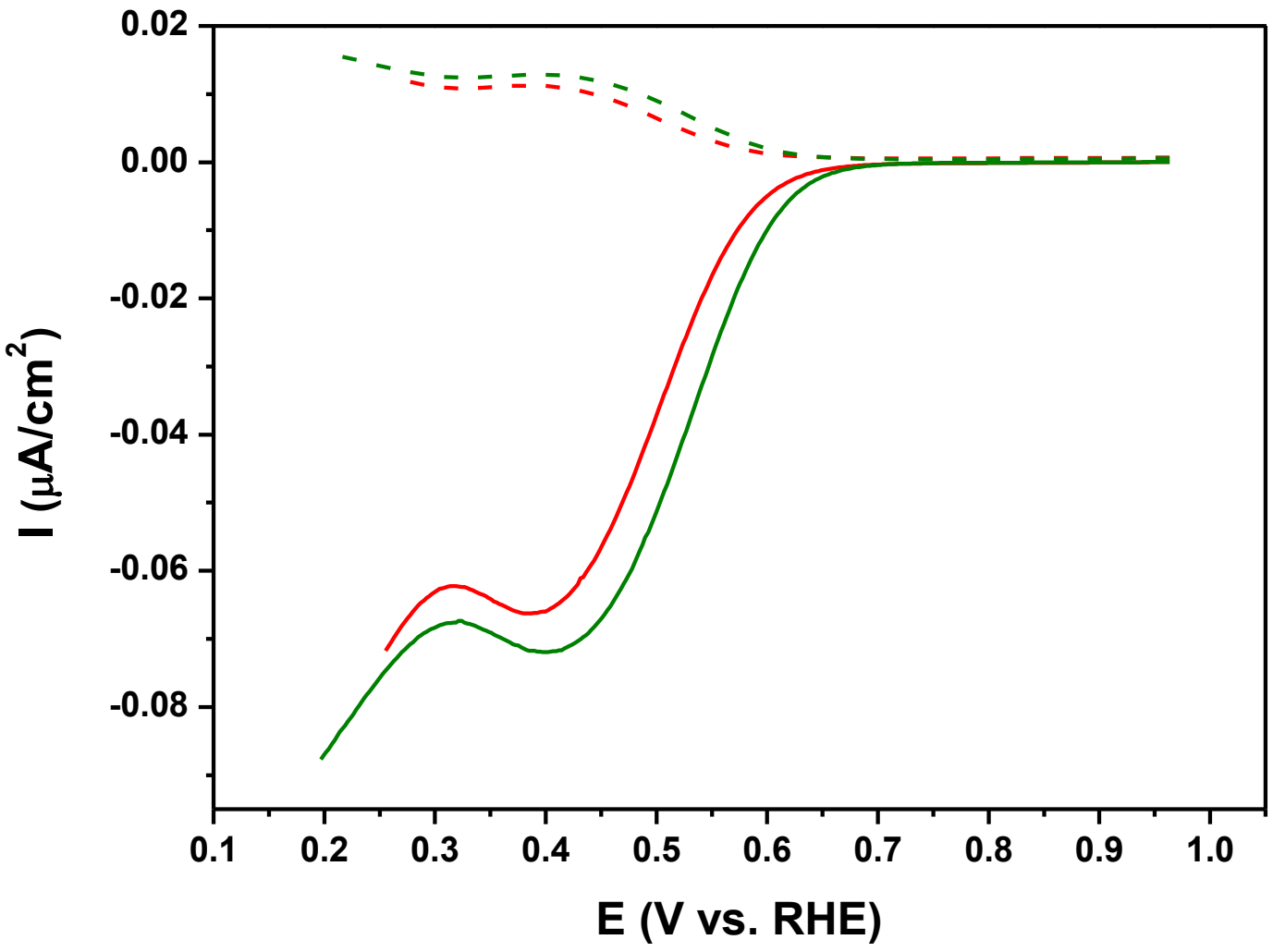

Figure S22. *ECSA-normalized current density (specific activity) for PAF-Phen-BT (green line) and PAF-Phen (red line).*

Table S4. *Non-pyrolyzed metal-free porous organic materials-based ORR.*

**Material**	**Electrolyte**	**Onset potential *vs.* NHE (V)**	**J (mA/cm^2)**	**Number of electrons**	**Additives**	**Reference**
**CTF**	0.1 M KOH	1.01	10	3.6-	Nafion	(11)
**PTM-CORF**	0.1 M KOH	0.80	-4.03	3.9-	Carbon Black	(12)
**JUC-528**	0.1 M KOH	0.70	-3.9	3.8	Acetylene black and Nafion	(13)
**TAPA-NDI-COF**	0.1 M KOH	0.70	-5.3	3.5	Carbon SuperP and Nafion	(14)
**TAPB-NDI-COF**		0.69	-4.9	3.5	Carbon SuperP and Nafion	
**TAPT-NDI-COF**		0.69	-4.5	3.5	Carbon SuperP and Nafion	
**NDI-COF**	0.1 M NaOH	0.65	-3.7	3.6	Non-additives	
**DAPT-TFP-COF**	0.1 M NaOH	0.79	-4.9	3.6	Non-additives	(15)
**DP-TPB**	0.1M KOH	0.83	-4.2	2.36	Ketjen black	(16)
**PAF-Phen**	0.1M KOH	0.61	-0.8	2.8	Non-additives	This work
**PAF-Phen-BT**		0.63	-1.2	2.7		

16. Computational models

Validation of cluster model

To validate the chosen cluster size, we compared the absorption spectra of PAF-Phen *n*-mers, (n = 1, 2, 4, 6, 8 and 10). The six molecules are shown in Figure S20. The first absorption band undergoes a blueshift as the system increases from 1 to 4 units, and reaches convergence at the tetramer (Figure S23). Larger clusters display essentially identical spectra, indicating that the tetramer is the smallest model able to reproduce the optical properties of the framework. On this basis and considering the substantially lower computational cost of the tetramer, this model was selected for the subsequent calculations on PAF-Phen and PAF-Phen-BT materials.

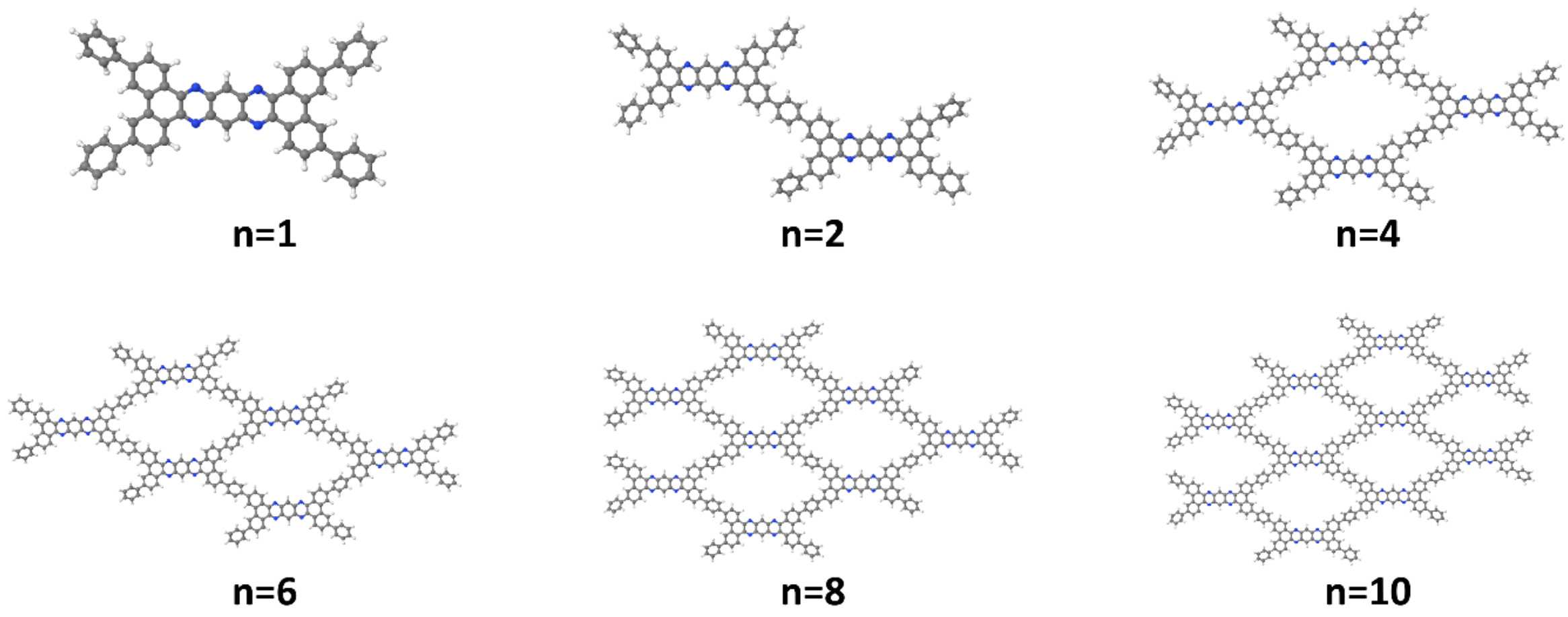

Figure S23. *PAF-Phen* n-*mer structures used to validate the cluster model.*

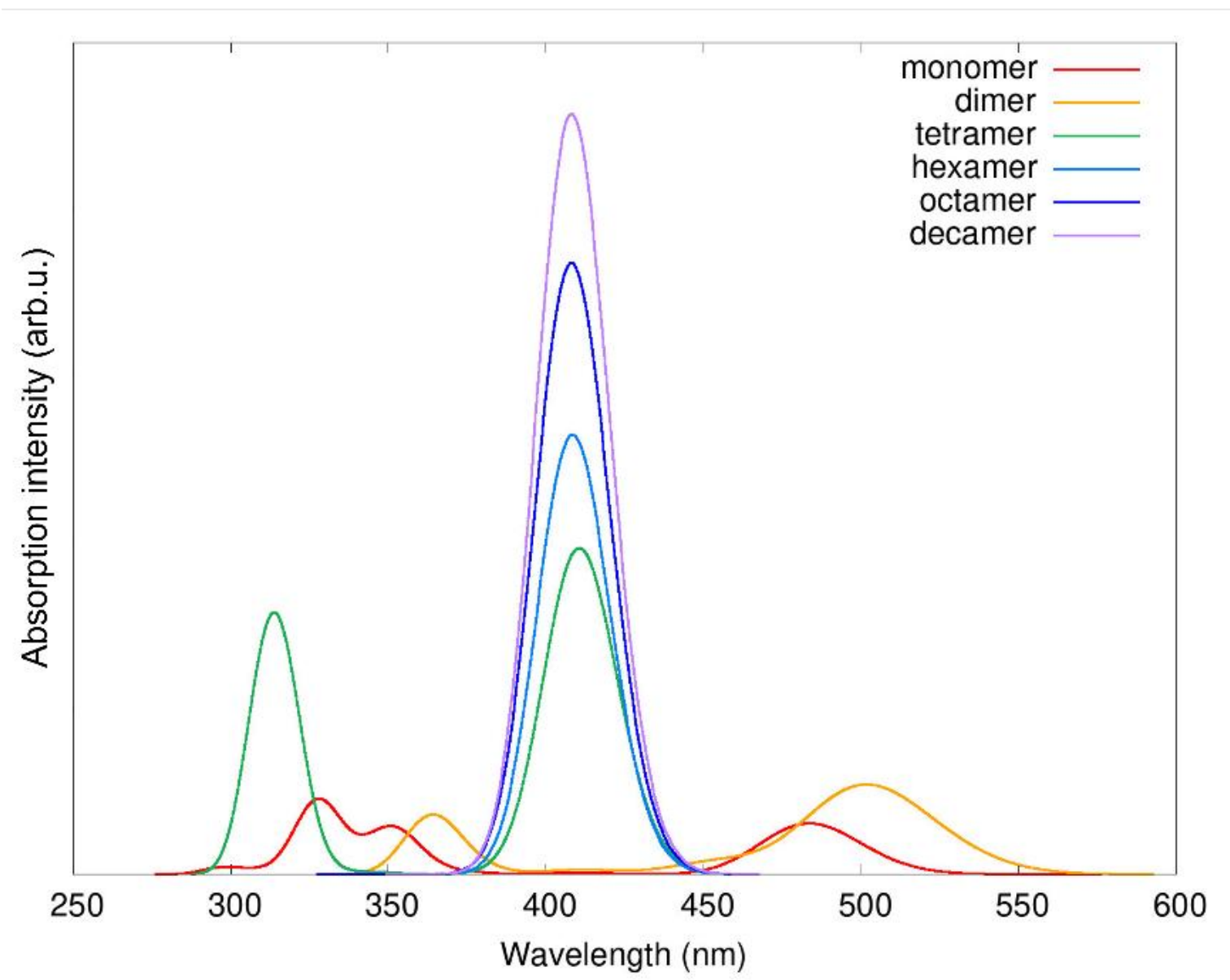

Figure S24. *Absorption spectra of the PAF-Phen n-mers at the TDA-CAM-B3LYP-D3BJ/def2-SVP level of theory in vacuum.*

Absorption spectra of the tetramers

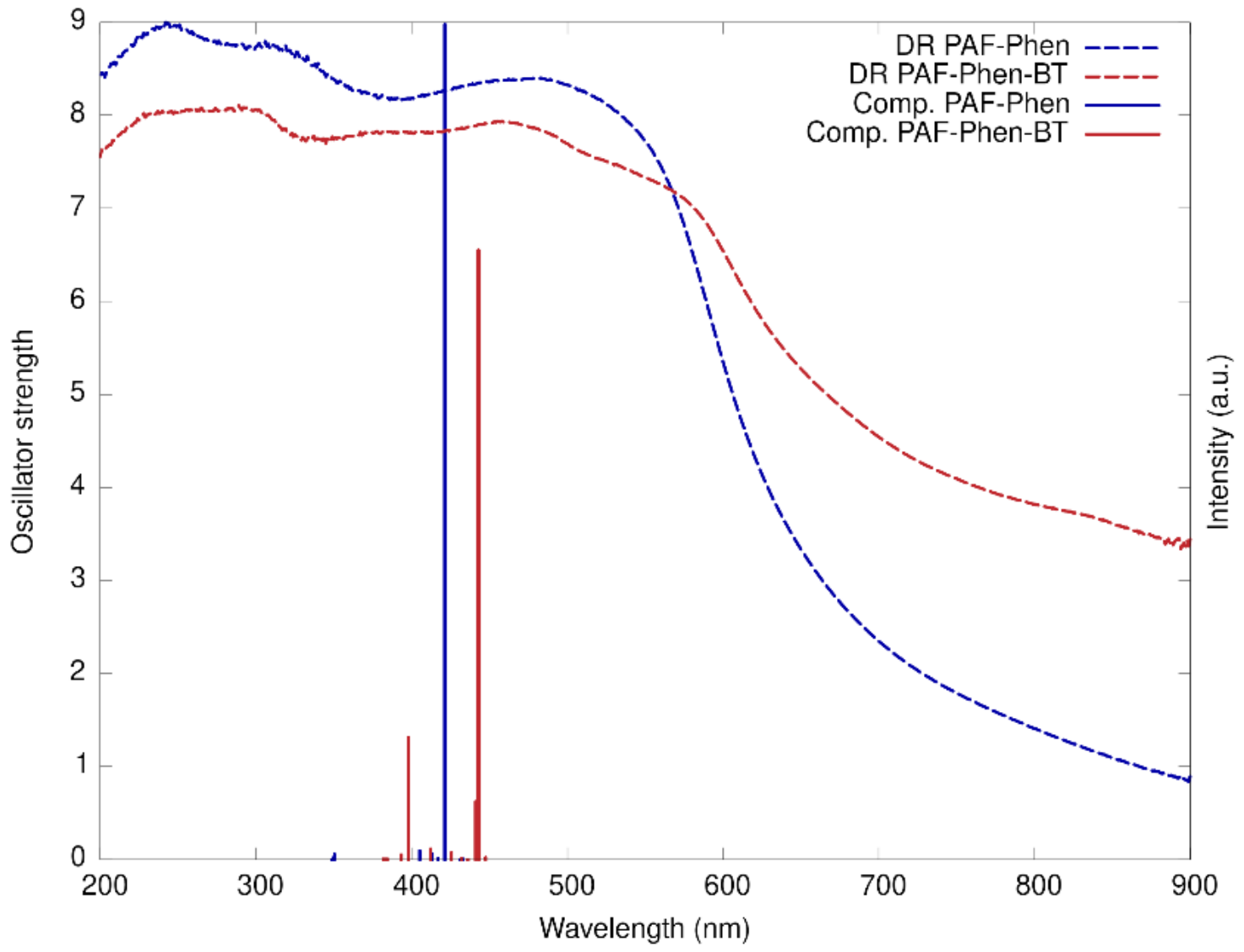

Figure S25. *Comparison between the experimental diffuse reflectance spectra (dashed lines) and the TD-CAM-B3LYP-D3BJ/def2-SVP calculated absorption spectra (vertical lines of the PAF-Phen and PAF-Phen-BT tetramers. The calculated spectra include the lowest 20 excited states of a single optimized tetramer model. The lowest-energy experimental absorption band is assigned to the calculated π→π* (S_1 and S_2) transitions discussed in the main text.*

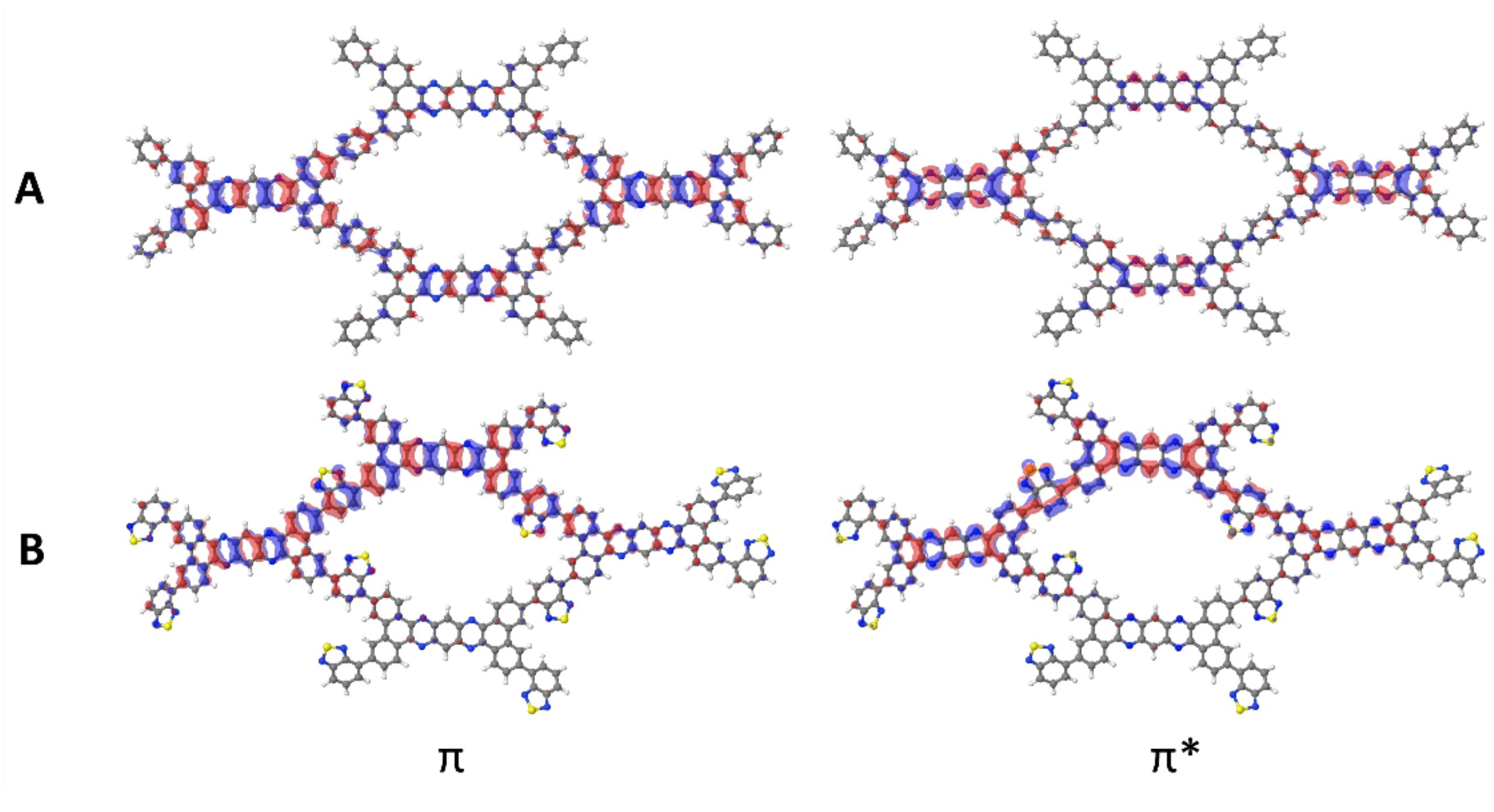

Figure S26. *Main natural transition orbitals involved in the bright excitations of (A) PAF-Phen and (B) PAF-Phen-BT tetramers at the Franck-Condon geometry, calculated at the TDA-CAM-B3LYP-D3BJ/def2-SVP level of theory in vacuum.*

Photosensitization mechanisms

We optimized the corresponding S_1 minima of both PAF tetramers. At these stationary points, we computed the spin-orbit coupling matrix elements between the S_1 and the lowest 30 triplet states to evaluate the efficiency of intersystem crossing (ISC). Table S5 reports these values together with the corresponding ($\Delta E(S_1\text{-}T_n)$) energy gaps and the $(H^{SO}(S_1\text{-}T_n)/\Delta E(S_1\text{-}T_n))^2$ ratios, as an estimate of ISC rates.

Table S5. *Spin-orbit coupling matrix elements between the S_1 and the triplets at the S1 minima of PAF-Phen and PAF-Phen-BT.*

	PAF-Phen				PAF-Phen-BT			
	E (eV)	$\Delta E(S_1\text{-}T_n)$ (eV)	$H^{SO}(S_1\text{-}T_n)$ (cm^{-1})	$(H^{SO}/\Delta E)^2$	E (eV)	$\Delta E(S_1\text{-}T_n)$ (eV)	$H^{SO}(S_1\text{-}T_n)$ (cm^{-1})	$(H^{SO}/\Delta E)^2$
S1	2.73	0.00	-	-	2.35	0.00	-	-
T1	1.44	-1.30	0.1	0.0	1.34	-1.02	0.0	0.0
T2	1.84	-0.90	0.0	0.0	1.57	-0.78	0.0	0.0
T3	1.84	-0.89	0.0	0.0	1.62	-0.74	0.0	0.0
T4	1.84	-0.89	0.0	0.0	1.65	-0.71	0.0	0.0
T5	2.29	-0.44	0.1	0.0	1.65	-0.70	0.0	0.0
T6	2.50	-0.23	0.0	0.0	1.98	-0.37	0.0	0.0
T7	2.51	-0.22	0.0	0.0	1.99	-0.36	0.0	0.0
T8	2.52	-0.22	0.0	0.0	2.02	-0.33	0.0	0.0
T9	2.64	-0.10	0.3	7.3	2.17	-0.18	0.0	0.0
T10	2.65	-0.08	1.5	331.3	2.17	-0.18	0.0	0.0
T11	2.66	-0.07	8.1	13895.8	2.19	-0.16	0.0	0.0
T12	2.77	0.04	0.1	2.9	2.20	-0.15	0.0	0.0
T13	2.77	0.04	0.0	0.5	2.23	-0.12	0.0	0.0
T14	2.77	0.04	0.0	0.1	2.24	-0.12	0.0	0.0
T15	2.79	0.06	0.2	7.0	2.24	-0.11	0.0	0.0
T16	2.79	0.06	0.1	3.8	2.25	-0.11	0.0	0.0
T17	2.79	0.06	0.1	3.1	2.30	-0.05	0.0	0.3
T18	2.80	0.06	0.0	0.4	2.33	-0.02	0.0	0.6
T19	2.81	0.07	0.1	1.5	2.38	0.03	0.0	0.1
T20	2.83	0.09	0.0	0.0	2.40	0.05	0.0	0.0
T21	2.89	0.16	0.2	1.9	2.55	0.20	1.4	51.4
T22	2.98	0.25	0.0	0.0	2.56	0.20	1.2	37.1
T23	3.01	0.28	0.0	0.0	2.58	0.22	1.5	42.7
T24	3.05	0.32	0.0	0.0	2.58	0.22	1.6	52.4
T25	3.07	0.34	0.0	0.0	2.59	0.23	0.0	0.0
T26	3.12	0.39	0.0	0.0	2.59	0.23	0.0	0.0
T27	3.14	0.41	0.0	0.0	2.59	0.24	0.1	0.2
T28	3.16	0.43	0.0	0.0	2.60	0.25	0.1	0.1
T29	3.33	0.60	0.1	0.0	2.60	0.25	0.1	0.2
T30	3.38	0.65	0.0	0.0	2.63	0.28	0.1	0.0

17. References

(1) Xue, H.; Xiong, S.; Mi, K.; Wang, Y. Visible-Light Degradation of Azo Dyes by Imine-Linked Covalent Organic Frameworks. *Green Energy Environ*. 2023, *8*, 194–199.

(2) Qi, W.; Wu, Q.; Wang, W.; Feng, J.; Su, Q. Fluorinated Covalent Organic Framework Materials for Photocatalytically Driven Benzylamine Coupling and Azo Dyes Degradation. *J. Photochem. Photobiol. A Chem*. 2023, *437*, 114502.

(3) Yang, Y.; Niu, H.; Xu, L.; Zhang, H.; Cai, Y. Triazine Functionalized Fully Conjugated Covalent Organic Framework for Efficient Photocatalysis. *Appl. Catal. B Environ*. 2020, *269*, 118799.

(4) He, S.; Yin, B.; Niu, H.; Cai, Y. Targeted Synthesis of Visible-Light-Driven Covalent Organic Framework Photocatalyst via Molecular Design and Precise Construction. *Appl. Catal. B Environ*. 2018, *239*, 147–153.

(5) Ponte, C.; Prieto, T.; López-Magano, A.; Moya, A.; Strutyński, K.; Estévez, L.; Fernandes, S.P.S.; Misa, I.; Sardo, M.; Rodriguez-Lorenzo, L.; Lebedev, O.I.; Kolen'ko, Y.V.; Melle-Franco, M.; Mas-Ballesté, R.; Salonen, L.M. Rapid Formation of Vinylene-Linked Covalent Organic Frameworks Promoted by Dipole Moment. *Chem. Mater.* 2025, *37*, 1923–1934.

(6) Xiao, Y-Z.; Mohammed, A.A.K.; Kuo, S-W.; EL-Mahdy, A.F.M. Innovative β-Ketoenamine-linked Covalent Organic Frameworks: Tailored D1-A-D2-A Structure for Highly Efficient Photocatalytic Degradation of Organic Pollutants. *Sep. Purif. Technol.* 2025, *356*, 129950.

(7) Shen, Y.; Hu, J.-Y.; Lu, L.; Zhu, C.; Fang, Q.-L.; Song, S. Enhanced Photocatalytic Performance of S-doped Covalent Triazine Framework for Organic Pollutant Degradation. *J. Zhejiang Univ-Sci A (Appl Phys & Eng)*, 2022, *23*, 988-997.

(8) Du, Y.; Ai, H.; Liu, Y.; Liu; H. A Novel Covalent Triazine-Based Frameworks as Photocatalysts for the Degradation of Dyes under Visible Light Irradiation. *Sustain. Energy Fuels.* 2023, *7*, 1747–1754.

(9) Mushtaq, S.; Bi, S.; Zhang, F.; Naseer; M.M. Fully Unsaturated All-Carbon Bifluorenylidene-Based Polymeric Frameworks: Synthesis and Efficient Photocatalysis. *New J. Chem.* 2022, *46*, 17374–17385.

(10) Xiao, Z.; Zhou, Y.; Xin, X.; Zhang, Q.; Zhang, L.; Wang, R.; Sun, D. Iron(III) Porphyrin-Based Porous Material as Photocatalyst for Highly Efficient and Selective Degradation of Congo Red. *Macromol. Chem. Phys*. 2016, *217*, 599-604.

(11) Liu, J.; Hu, Y.; Cao. J. Covalent Triazine-Based Frameworks as Efficient Metal-Free Electrocatalysts for Oxygen Reduction Reaction in Alkaline Media. *Catal. Commun.* 2015, *66*, 91.

(12) Wu, S.; Li, M.; Phan, H.; Wang, D.; Herng, T.S.; Ding, J.; Lu, Z.; Wu, J. Toward Two-Dimensional π-Conjugated Covalent Organic Radical Frameworks. *Angew. Chem. Int. Ed.* 2018, *57*, 8007.

(13) Li, D.; Li, C.; Zhang, L.; Li, H.; Zhu, L.; Yang, D.; Fang, Q.; Qiu, S.; Yao, X. Metal-Free Thiophene-Sulfur Covalent Organic Frameworks: Precise and Controllable Synthesis of Catalytic Active Sites for Oxygen Reduction. *J. Am. Chem. Soc.* 2020, *142*, 8104.

(14) Martínez-Fernández, M.; Martínez-Periñán, E.; Royuela, S.; Martínez, J.I.; Zamora, F.; Lorenzo, E.; Segura, J.L. Covalent Organic Frameworks Based on Electroactive Naphthalenediimide as Active Electrocatalysts toward Oxygen Reduction Reaction. *Appl. Mater. Today* 2022, *26*, 101384.

(15) García-Arroyo, P.; Martínez-Periñán, E.; Cabrera-Trujillo, J.J.; Salagre, E.; Michel, E.G.; Martínez, J.I.; Lorenzo, E.; Segura, J.L. Pyrenetetraone-based Covalent Organic Framework as an Effective Electrocatalyst for Oxygen Reduction Reaction. *Nano Res.* 2022, *15*, 3907.

(16) Wei, S.; Wu, X.; Zhu, S.; Wang, Z.; Wang, J.; Lu, C.-Z.; Xie, Y. Efficient Oxygen Reduction through Metal-free 3D Covalent Organic Frameworks: a Novel Approach. *Sci. China Mater.* 2024, *67*, 3589.
